# Supplementary figures and images for: Enhancing Alzheimer’s disease classification through split federated learning and GANs for imbalanced datasets (part 2 of 4)
Source: PeerJ Comput Sci. 2024 Nov 29;10:e2459. doi: 10.7717/peerj-cs.2459 (PMC11623002; doi:10.7717/peerj-cs.2459)

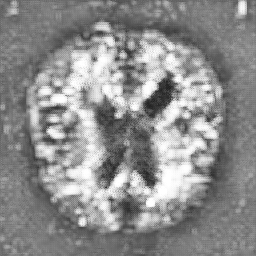

Supplement: Supplemental Information 1 — Image source: https://www.kaggle.com/datasets/tourist55/alzheimers-dataset-4-class-of-images. License: Open Database License (ODbL) v1.0. [file peerj-cs-10-2459-s001.zip › case4/test1/ModerateDemented/generated_image_96.png]

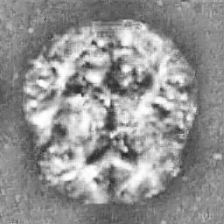

Supplement: Supplemental Information 1 — Image source: https://www.kaggle.com/datasets/tourist55/alzheimers-dataset-4-class-of-images. License: Open Database License (ODbL) v1.0. [file peerj-cs-10-2459-s001.zip › case4/test1/MildDemented/Copy of Copy of generated_image_class_1_5.png]

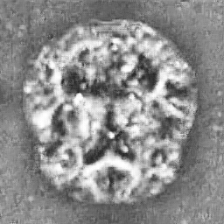

Supplement: Supplemental Information 1 — Image source: https://www.kaggle.com/datasets/tourist55/alzheimers-dataset-4-class-of-images. License: Open Database License (ODbL) v1.0. [file peerj-cs-10-2459-s001.zip › case4/test1/MildDemented/Copy of Copy of generated_image_class_1_12.png]

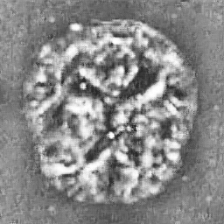

Supplement: Supplemental Information 1 — Image source: https://www.kaggle.com/datasets/tourist55/alzheimers-dataset-4-class-of-images. License: Open Database License (ODbL) v1.0. [file peerj-cs-10-2459-s001.zip › case4/test1/MildDemented/Copy of Copy of generated_image_class_1_32.png]

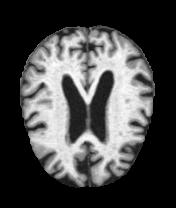

Supplement: Supplemental Information 1 — Image source: https://www.kaggle.com/datasets/tourist55/alzheimers-dataset-4-class-of-images. License: Open Database License (ODbL) v1.0. [file peerj-cs-10-2459-s001.zip › case4/test1/MildDemented/Copy of 28 (19).jpg]

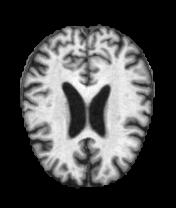

Supplement: Supplemental Information 1 — Image source: https://www.kaggle.com/datasets/tourist55/alzheimers-dataset-4-class-of-images. License: Open Database License (ODbL) v1.0. [file peerj-cs-10-2459-s001.zip › case4/test1/MildDemented/Copy of 27 (16).jpg]

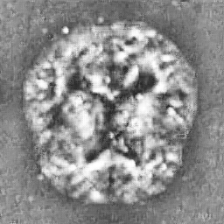

Supplement: Supplemental Information 1 — Image source: https://www.kaggle.com/datasets/tourist55/alzheimers-dataset-4-class-of-images. License: Open Database License (ODbL) v1.0. [file peerj-cs-10-2459-s001.zip › case4/test1/MildDemented/Copy of Copy of generated_image_class_1_6.png]

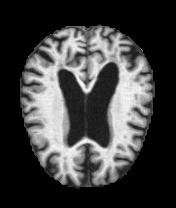

Supplement: Supplemental Information 1 — Image source: https://www.kaggle.com/datasets/tourist55/alzheimers-dataset-4-class-of-images. License: Open Database License (ODbL) v1.0. [file peerj-cs-10-2459-s001.zip › case4/test1/MildDemented/Copy of 27 (10).jpg]

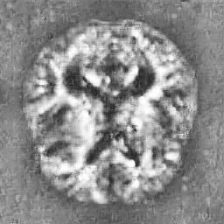

Supplement: Supplemental Information 1 — Image source: https://www.kaggle.com/datasets/tourist55/alzheimers-dataset-4-class-of-images. License: Open Database License (ODbL) v1.0. [file peerj-cs-10-2459-s001.zip › case4/test1/MildDemented/Copy of Copy of generated_image_class_1_2.png]

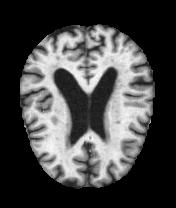

Supplement: Supplemental Information 1 — Image source: https://www.kaggle.com/datasets/tourist55/alzheimers-dataset-4-class-of-images. License: Open Database License (ODbL) v1.0. [file peerj-cs-10-2459-s001.zip › case4/test1/MildDemented/Copy of 26 (26).jpg]

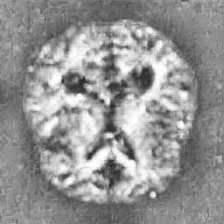

Supplement: Supplemental Information 1 — Image source: https://www.kaggle.com/datasets/tourist55/alzheimers-dataset-4-class-of-images. License: Open Database License (ODbL) v1.0. [file peerj-cs-10-2459-s001.zip › case4/test1/MildDemented/Copy of Copy of generated_image_class_1_28.png]

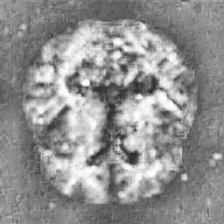

Supplement: Supplemental Information 1 — Image source: https://www.kaggle.com/datasets/tourist55/alzheimers-dataset-4-class-of-images. License: Open Database License (ODbL) v1.0. [file peerj-cs-10-2459-s001.zip › case4/test1/MildDemented/Copy of Copy of generated_image_class_1_7.png]

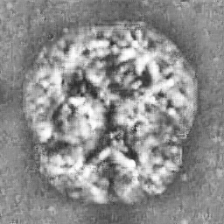

Supplement: Supplemental Information 1 — Image source: https://www.kaggle.com/datasets/tourist55/alzheimers-dataset-4-class-of-images. License: Open Database License (ODbL) v1.0. [file peerj-cs-10-2459-s001.zip › case4/test1/MildDemented/Copy of Copy of generated_image_class_1_27.png]

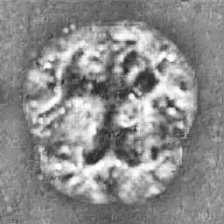

Supplement: Supplemental Information 1 — Image source: https://www.kaggle.com/datasets/tourist55/alzheimers-dataset-4-class-of-images. License: Open Database License (ODbL) v1.0. [file peerj-cs-10-2459-s001.zip › case4/test1/MildDemented/Copy of Copy of generated_image_class_1_1.png]

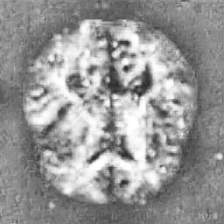

Supplement: Supplemental Information 1 — Image source: https://www.kaggle.com/datasets/tourist55/alzheimers-dataset-4-class-of-images. License: Open Database License (ODbL) v1.0. [file peerj-cs-10-2459-s001.zip › case4/test1/MildDemented/Copy of Copy of generated_image_class_1_8.png]

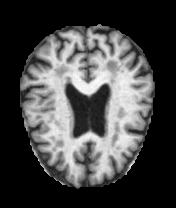

Supplement: Supplemental Information 1 — Image source: https://www.kaggle.com/datasets/tourist55/alzheimers-dataset-4-class-of-images. License: Open Database License (ODbL) v1.0. [file peerj-cs-10-2459-s001.zip › case4/test1/MildDemented/Copy of 27 (5).jpg]

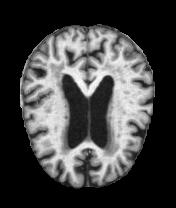

Supplement: Supplemental Information 1 — Image source: https://www.kaggle.com/datasets/tourist55/alzheimers-dataset-4-class-of-images. License: Open Database License (ODbL) v1.0. [file peerj-cs-10-2459-s001.zip › case4/test1/MildDemented/Copy of 26 (20).jpg]

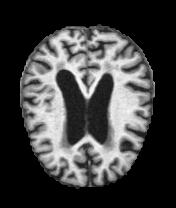

Supplement: Supplemental Information 1 — Image source: https://www.kaggle.com/datasets/tourist55/alzheimers-dataset-4-class-of-images. License: Open Database License (ODbL) v1.0. [file peerj-cs-10-2459-s001.zip › case4/test1/MildDemented/Copy of 27 (11).jpg]

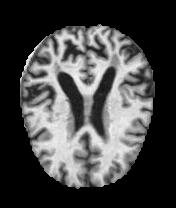

Supplement: Supplemental Information 1 — Image source: https://www.kaggle.com/datasets/tourist55/alzheimers-dataset-4-class-of-images. License: Open Database License (ODbL) v1.0. [file peerj-cs-10-2459-s001.zip › case4/test1/MildDemented/Copy of 27 (13).jpg]

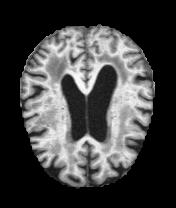

Supplement: Supplemental Information 1 — Image source: https://www.kaggle.com/datasets/tourist55/alzheimers-dataset-4-class-of-images. License: Open Database License (ODbL) v1.0. [file peerj-cs-10-2459-s001.zip › case4/test1/MildDemented/Copy of 27 (24).jpg]

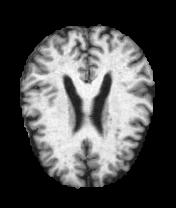

Supplement: Supplemental Information 1 — Image source: https://www.kaggle.com/datasets/tourist55/alzheimers-dataset-4-class-of-images. License: Open Database License (ODbL) v1.0. [file peerj-cs-10-2459-s001.zip › case4/test1/MildDemented/Copy of 27 (18).jpg]

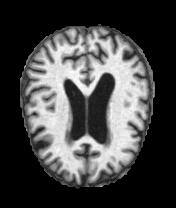

Supplement: Supplemental Information 1 — Image source: https://www.kaggle.com/datasets/tourist55/alzheimers-dataset-4-class-of-images. License: Open Database License (ODbL) v1.0. [file peerj-cs-10-2459-s001.zip › case4/test1/MildDemented/Copy of 27 (12).jpg]

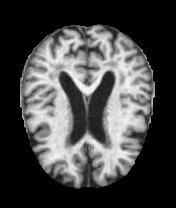

Supplement: Supplemental Information 1 — Image source: https://www.kaggle.com/datasets/tourist55/alzheimers-dataset-4-class-of-images. License: Open Database License (ODbL) v1.0. [file peerj-cs-10-2459-s001.zip › case4/test1/MildDemented/Copy of 26.jpg]

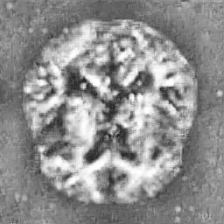

Supplement: Supplemental Information 1 — Image source: https://www.kaggle.com/datasets/tourist55/alzheimers-dataset-4-class-of-images. License: Open Database License (ODbL) v1.0. [file peerj-cs-10-2459-s001.zip › case4/test1/MildDemented/Copy of Copy of generated_image_class_1_9.png]

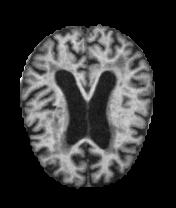

Supplement: Supplemental Information 1 — Image source: https://www.kaggle.com/datasets/tourist55/alzheimers-dataset-4-class-of-images. License: Open Database License (ODbL) v1.0. [file peerj-cs-10-2459-s001.zip › case4/test1/MildDemented/Copy of 27 (14).jpg]

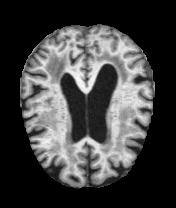

Supplement: Supplemental Information 1 — Image source: https://www.kaggle.com/datasets/tourist55/alzheimers-dataset-4-class-of-images. License: Open Database License (ODbL) v1.0. [file peerj-cs-10-2459-s001.zip › case4/test1/MildDemented/Copy of 26 (24).jpg]

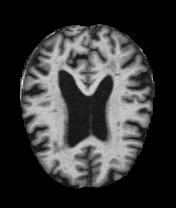

Supplement: Supplemental Information 1 — Image source: https://www.kaggle.com/datasets/tourist55/alzheimers-dataset-4-class-of-images. License: Open Database License (ODbL) v1.0. [file peerj-cs-10-2459-s001.zip › case4/test1/MildDemented/Copy of 26 (27).jpg]

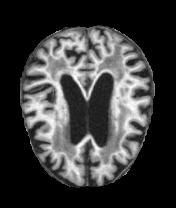

Supplement: Supplemental Information 1 — Image source: https://www.kaggle.com/datasets/tourist55/alzheimers-dataset-4-class-of-images. License: Open Database License (ODbL) v1.0. [file peerj-cs-10-2459-s001.zip › case4/test1/MildDemented/Copy of 28 (2).jpg]

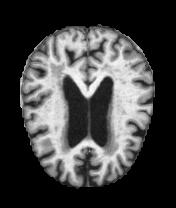

Supplement: Supplemental Information 1 — Image source: https://www.kaggle.com/datasets/tourist55/alzheimers-dataset-4-class-of-images. License: Open Database License (ODbL) v1.0. [file peerj-cs-10-2459-s001.zip › case4/test1/MildDemented/Copy of 27 (20).jpg]

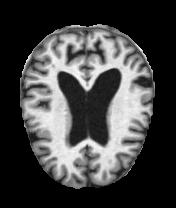

Supplement: Supplemental Information 1 — Image source: https://www.kaggle.com/datasets/tourist55/alzheimers-dataset-4-class-of-images. License: Open Database License (ODbL) v1.0. [file peerj-cs-10-2459-s001.zip › case4/test1/MildDemented/Copy of 26 (21).jpg]

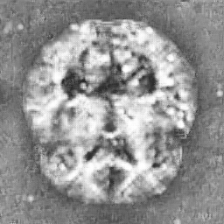

Supplement: Supplemental Information 1 — Image source: https://www.kaggle.com/datasets/tourist55/alzheimers-dataset-4-class-of-images. License: Open Database License (ODbL) v1.0. [file peerj-cs-10-2459-s001.zip › case4/test1/MildDemented/Copy of Copy of generated_image_class_1_17.png]

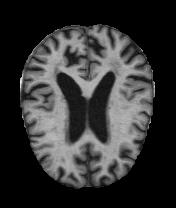

Supplement: Supplemental Information 1 — Image source: https://www.kaggle.com/datasets/tourist55/alzheimers-dataset-4-class-of-images. License: Open Database License (ODbL) v1.0. [file peerj-cs-10-2459-s001.zip › case4/test1/MildDemented/Copy of 26 (23).jpg]

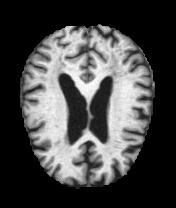

Supplement: Supplemental Information 1 — Image source: https://www.kaggle.com/datasets/tourist55/alzheimers-dataset-4-class-of-images. License: Open Database License (ODbL) v1.0. [file peerj-cs-10-2459-s001.zip › case4/test1/MildDemented/Copy of 27 (25).jpg]

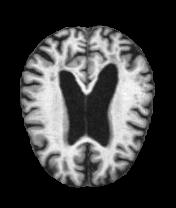

Supplement: Supplemental Information 1 — Image source: https://www.kaggle.com/datasets/tourist55/alzheimers-dataset-4-class-of-images. License: Open Database License (ODbL) v1.0. [file peerj-cs-10-2459-s001.zip › case4/test1/MildDemented/Copy of 28 (10).jpg]

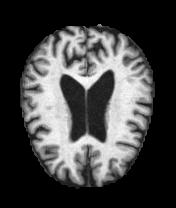

Supplement: Supplemental Information 1 — Image source: https://www.kaggle.com/datasets/tourist55/alzheimers-dataset-4-class-of-images. License: Open Database License (ODbL) v1.0. [file peerj-cs-10-2459-s001.zip › case4/test1/MildDemented/Copy of 27 (3).jpg]

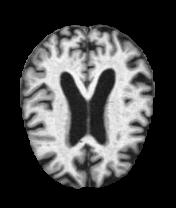

Supplement: Supplemental Information 1 — Image source: https://www.kaggle.com/datasets/tourist55/alzheimers-dataset-4-class-of-images. License: Open Database License (ODbL) v1.0. [file peerj-cs-10-2459-s001.zip › case4/test1/MildDemented/Copy of 26 (19).jpg]

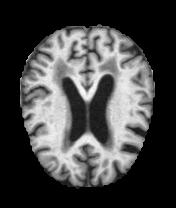

Supplement: Supplemental Information 1 — Image source: https://www.kaggle.com/datasets/tourist55/alzheimers-dataset-4-class-of-images. License: Open Database License (ODbL) v1.0. [file peerj-cs-10-2459-s001.zip › case4/test1/MildDemented/Copy of 27 (22).jpg]

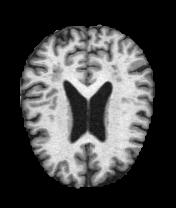

Supplement: Supplemental Information 1 — Image source: https://www.kaggle.com/datasets/tourist55/alzheimers-dataset-4-class-of-images. License: Open Database License (ODbL) v1.0. [file peerj-cs-10-2459-s001.zip › case4/test1/MildDemented/Copy of 27 (28).jpg]

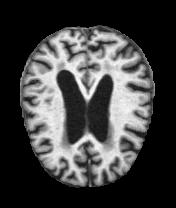

Supplement: Supplemental Information 1 — Image source: https://www.kaggle.com/datasets/tourist55/alzheimers-dataset-4-class-of-images. License: Open Database License (ODbL) v1.0. [file peerj-cs-10-2459-s001.zip › case4/test1/MildDemented/Copy of 28 (11).jpg]

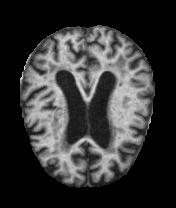

Supplement: Supplemental Information 1 — Image source: https://www.kaggle.com/datasets/tourist55/alzheimers-dataset-4-class-of-images. License: Open Database License (ODbL) v1.0. [file peerj-cs-10-2459-s001.zip › case4/test1/MildDemented/Copy of 28 (14).jpg]

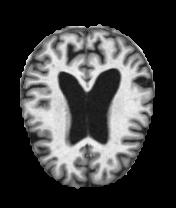

Supplement: Supplemental Information 1 — Image source: https://www.kaggle.com/datasets/tourist55/alzheimers-dataset-4-class-of-images. License: Open Database License (ODbL) v1.0. [file peerj-cs-10-2459-s001.zip › case4/test1/MildDemented/Copy of 27 (21).jpg]

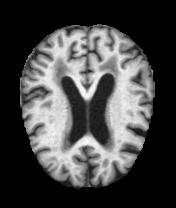

Supplement: Supplemental Information 1 — Image source: https://www.kaggle.com/datasets/tourist55/alzheimers-dataset-4-class-of-images. License: Open Database License (ODbL) v1.0. [file peerj-cs-10-2459-s001.zip › case4/test1/MildDemented/Copy of 26 (22).jpg]

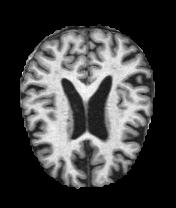

Supplement: Supplemental Information 1 — Image source: https://www.kaggle.com/datasets/tourist55/alzheimers-dataset-4-class-of-images. License: Open Database License (ODbL) v1.0. [file peerj-cs-10-2459-s001.zip › case4/test1/MildDemented/Copy of 27 (15).jpg]

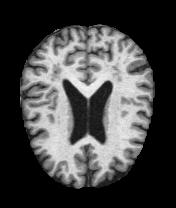

Supplement: Supplemental Information 1 — Image source: https://www.kaggle.com/datasets/tourist55/alzheimers-dataset-4-class-of-images. License: Open Database License (ODbL) v1.0. [file peerj-cs-10-2459-s001.zip › case4/test1/MildDemented/Copy of 26 (28).jpg]

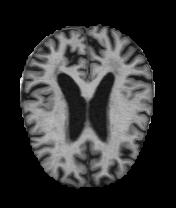

Supplement: Supplemental Information 1 — Image source: https://www.kaggle.com/datasets/tourist55/alzheimers-dataset-4-class-of-images. License: Open Database License (ODbL) v1.0. [file peerj-cs-10-2459-s001.zip › case4/test1/MildDemented/Copy of 27 (23).jpg]

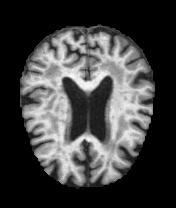

Supplement: Supplemental Information 1 — Image source: https://www.kaggle.com/datasets/tourist55/alzheimers-dataset-4-class-of-images. License: Open Database License (ODbL) v1.0. [file peerj-cs-10-2459-s001.zip › case4/test1/MildDemented/Copy of 27 (17).jpg]

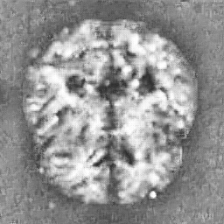

Supplement: Supplemental Information 1 — Image source: https://www.kaggle.com/datasets/tourist55/alzheimers-dataset-4-class-of-images. License: Open Database License (ODbL) v1.0. [file peerj-cs-10-2459-s001.zip › case4/test1/MildDemented/Copy of Copy of generated_image_class_1_10.png]

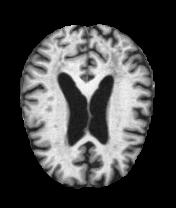

Supplement: Supplemental Information 1 — Image source: https://www.kaggle.com/datasets/tourist55/alzheimers-dataset-4-class-of-images. License: Open Database License (ODbL) v1.0. [file peerj-cs-10-2459-s001.zip › case4/test1/MildDemented/Copy of 26 (25).jpg]

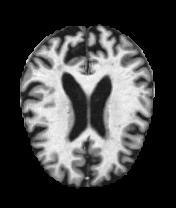

Supplement: Supplemental Information 1 — Image source: https://www.kaggle.com/datasets/tourist55/alzheimers-dataset-4-class-of-images. License: Open Database License (ODbL) v1.0. [file peerj-cs-10-2459-s001.zip › case4/test1/MildDemented/Copy of 27 (9).jpg]

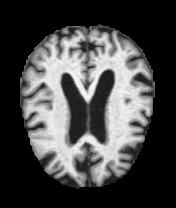

Supplement: Supplemental Information 1 — Image source: https://www.kaggle.com/datasets/tourist55/alzheimers-dataset-4-class-of-images. License: Open Database License (ODbL) v1.0. [file peerj-cs-10-2459-s001.zip › case4/test1/MildDemented/Copy of 27 (19).jpg]

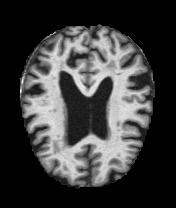

Supplement: Supplemental Information 1 — Image source: https://www.kaggle.com/datasets/tourist55/alzheimers-dataset-4-class-of-images. License: Open Database License (ODbL) v1.0. [file peerj-cs-10-2459-s001.zip › case4/test1/MildDemented/Copy of 27 (27).jpg]

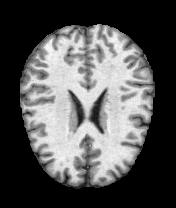

Supplement: Supplemental Information 1 — Image source: https://www.kaggle.com/datasets/tourist55/alzheimers-dataset-4-class-of-images. License: Open Database License (ODbL) v1.0. [file peerj-cs-10-2459-s001.zip › case4/test1/NonDemented/Copy of 26 (86).jpg]

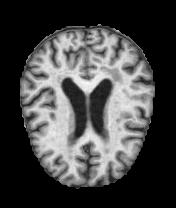

Supplement: Supplemental Information 1 — Image source: https://www.kaggle.com/datasets/tourist55/alzheimers-dataset-4-class-of-images. License: Open Database License (ODbL) v1.0. [file peerj-cs-10-2459-s001.zip › case4/test1/MildDemented/Copy of 27 (4).jpg]

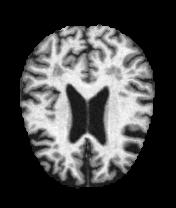

Supplement: Supplemental Information 1 — Image source: https://www.kaggle.com/datasets/tourist55/alzheimers-dataset-4-class-of-images. License: Open Database License (ODbL) v1.0. [file peerj-cs-10-2459-s001.zip › case4/test1/MildDemented/Copy of 27 (7).jpg]

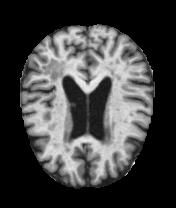

Supplement: Supplemental Information 1 — Image source: https://www.kaggle.com/datasets/tourist55/alzheimers-dataset-4-class-of-images. License: Open Database License (ODbL) v1.0. [file peerj-cs-10-2459-s001.zip › case4/test1/NonDemented/Copy of 26 (65).jpg]

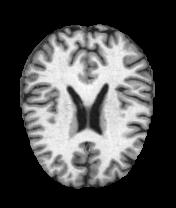

Supplement: Supplemental Information 1 — Image source: https://www.kaggle.com/datasets/tourist55/alzheimers-dataset-4-class-of-images. License: Open Database License (ODbL) v1.0. [file peerj-cs-10-2459-s001.zip › case4/test1/NonDemented/Copy of 26 (76).jpg]

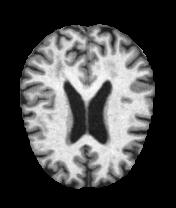

Supplement: Supplemental Information 1 — Image source: https://www.kaggle.com/datasets/tourist55/alzheimers-dataset-4-class-of-images. License: Open Database License (ODbL) v1.0. [file peerj-cs-10-2459-s001.zip › case4/test1/NonDemented/Copy of 26 (80).jpg]

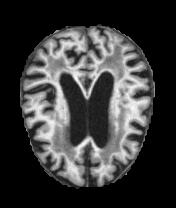

Supplement: Supplemental Information 1 — Image source: https://www.kaggle.com/datasets/tourist55/alzheimers-dataset-4-class-of-images. License: Open Database License (ODbL) v1.0. [file peerj-cs-10-2459-s001.zip › case4/test1/MildDemented/Copy of 27 (2).jpg]

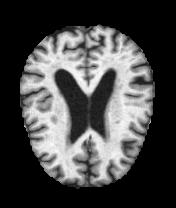

Supplement: Supplemental Information 1 — Image source: https://www.kaggle.com/datasets/tourist55/alzheimers-dataset-4-class-of-images. License: Open Database License (ODbL) v1.0. [file peerj-cs-10-2459-s001.zip › case4/test1/MildDemented/Copy of 27 (26).jpg]

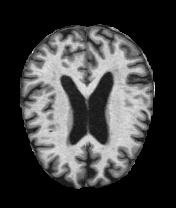

Supplement: Supplemental Information 1 — Image source: https://www.kaggle.com/datasets/tourist55/alzheimers-dataset-4-class-of-images. License: Open Database License (ODbL) v1.0. [file peerj-cs-10-2459-s001.zip › case4/test1/NonDemented/Copy of 26 (75).jpg]

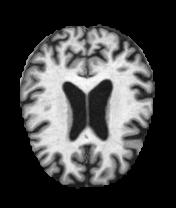

Supplement: Supplemental Information 1 — Image source: https://www.kaggle.com/datasets/tourist55/alzheimers-dataset-4-class-of-images. License: Open Database License (ODbL) v1.0. [file peerj-cs-10-2459-s001.zip › case4/test1/MildDemented/Copy of 27 (6).jpg]

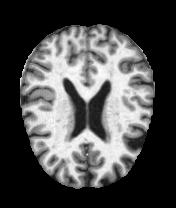

Supplement: Supplemental Information 1 — Image source: https://www.kaggle.com/datasets/tourist55/alzheimers-dataset-4-class-of-images. License: Open Database License (ODbL) v1.0. [file peerj-cs-10-2459-s001.zip › case4/test1/NonDemented/Copy of 26 (63).jpg]

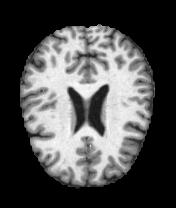

Supplement: Supplemental Information 1 — Image source: https://www.kaggle.com/datasets/tourist55/alzheimers-dataset-4-class-of-images. License: Open Database License (ODbL) v1.0. [file peerj-cs-10-2459-s001.zip › case4/test1/NonDemented/Copy of 27 (22).jpg]

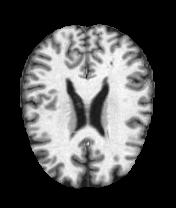

Supplement: Supplemental Information 1 — Image source: https://www.kaggle.com/datasets/tourist55/alzheimers-dataset-4-class-of-images. License: Open Database License (ODbL) v1.0. [file peerj-cs-10-2459-s001.zip › case4/test1/NonDemented/Copy of 26 (67).jpg]

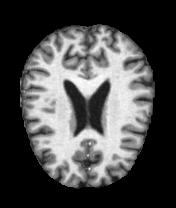

Supplement: Supplemental Information 1 — Image source: https://www.kaggle.com/datasets/tourist55/alzheimers-dataset-4-class-of-images. License: Open Database License (ODbL) v1.0. [file peerj-cs-10-2459-s001.zip › case4/test1/NonDemented/Copy of 26 (100).jpg]

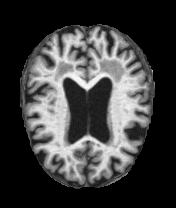

Supplement: Supplemental Information 1 — Image source: https://www.kaggle.com/datasets/tourist55/alzheimers-dataset-4-class-of-images. License: Open Database License (ODbL) v1.0. [file peerj-cs-10-2459-s001.zip › case4/test1/NonDemented/Copy of 26 (64).jpg]

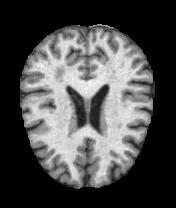

Supplement: Supplemental Information 1 — Image source: https://www.kaggle.com/datasets/tourist55/alzheimers-dataset-4-class-of-images. License: Open Database License (ODbL) v1.0. [file peerj-cs-10-2459-s001.zip › case4/test1/NonDemented/Copy of 26 (81).jpg]

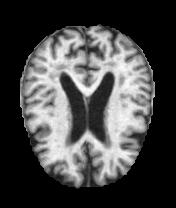

Supplement: Supplemental Information 1 — Image source: https://www.kaggle.com/datasets/tourist55/alzheimers-dataset-4-class-of-images. License: Open Database License (ODbL) v1.0. [file peerj-cs-10-2459-s001.zip › case4/test1/MildDemented/Copy of 27.jpg]

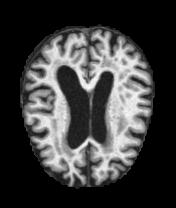

Supplement: Supplemental Information 1 — Image source: https://www.kaggle.com/datasets/tourist55/alzheimers-dataset-4-class-of-images. License: Open Database License (ODbL) v1.0. [file peerj-cs-10-2459-s001.zip › case4/test1/MildDemented/Copy of 27 (8).jpg]

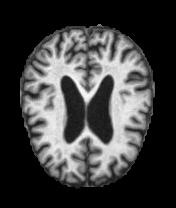

Supplement: Supplemental Information 1 — Image source: https://www.kaggle.com/datasets/tourist55/alzheimers-dataset-4-class-of-images. License: Open Database License (ODbL) v1.0. [file peerj-cs-10-2459-s001.zip › case4/test1/NonDemented/Copy of 27 (11).jpg]

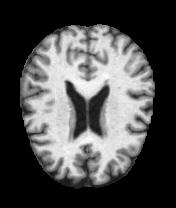

Supplement: Supplemental Information 1 — Image source: https://www.kaggle.com/datasets/tourist55/alzheimers-dataset-4-class-of-images. License: Open Database License (ODbL) v1.0. [file peerj-cs-10-2459-s001.zip › case4/test1/NonDemented/Copy of 26 (62).jpg]

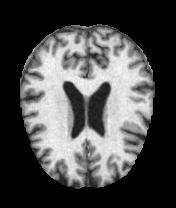

Supplement: Supplemental Information 1 — Image source: https://www.kaggle.com/datasets/tourist55/alzheimers-dataset-4-class-of-images. License: Open Database License (ODbL) v1.0. [file peerj-cs-10-2459-s001.zip › case4/test1/NonDemented/Copy of 26 (69).jpg]

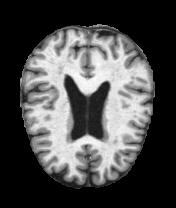

Supplement: Supplemental Information 1 — Image source: https://www.kaggle.com/datasets/tourist55/alzheimers-dataset-4-class-of-images. License: Open Database License (ODbL) v1.0. [file peerj-cs-10-2459-s001.zip › case4/test1/NonDemented/Copy of 26 (90).jpg]

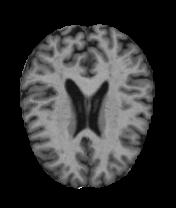

Supplement: Supplemental Information 1 — Image source: https://www.kaggle.com/datasets/tourist55/alzheimers-dataset-4-class-of-images. License: Open Database License (ODbL) v1.0. [file peerj-cs-10-2459-s001.zip › case4/test1/NonDemented/Copy of 26 (97).jpg]

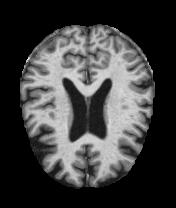

Supplement: Supplemental Information 1 — Image source: https://www.kaggle.com/datasets/tourist55/alzheimers-dataset-4-class-of-images. License: Open Database License (ODbL) v1.0. [file peerj-cs-10-2459-s001.zip › case4/test1/NonDemented/Copy of 26 (73).jpg]

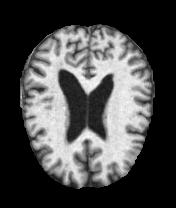

Supplement: Supplemental Information 1 — Image source: https://www.kaggle.com/datasets/tourist55/alzheimers-dataset-4-class-of-images. License: Open Database License (ODbL) v1.0. [file peerj-cs-10-2459-s001.zip › case4/test1/NonDemented/Copy of 26 (92).jpg]

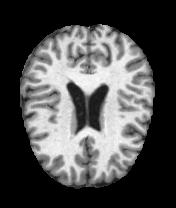

Supplement: Supplemental Information 1 — Image source: https://www.kaggle.com/datasets/tourist55/alzheimers-dataset-4-class-of-images. License: Open Database License (ODbL) v1.0. [file peerj-cs-10-2459-s001.zip › case4/test1/NonDemented/Copy of 26 (66).jpg]

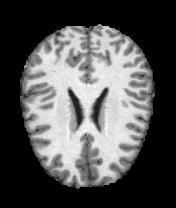

Supplement: Supplemental Information 1 — Image source: https://www.kaggle.com/datasets/tourist55/alzheimers-dataset-4-class-of-images. License: Open Database License (ODbL) v1.0. [file peerj-cs-10-2459-s001.zip › case4/test1/NonDemented/Copy of 27 (14).jpg]

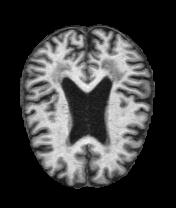

Supplement: Supplemental Information 1 — Image source: https://www.kaggle.com/datasets/tourist55/alzheimers-dataset-4-class-of-images. License: Open Database License (ODbL) v1.0. [file peerj-cs-10-2459-s001.zip › case4/test1/NonDemented/Copy of 26 (74).jpg]

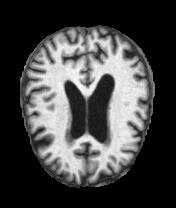

Supplement: Supplemental Information 1 — Image source: https://www.kaggle.com/datasets/tourist55/alzheimers-dataset-4-class-of-images. License: Open Database License (ODbL) v1.0. [file peerj-cs-10-2459-s001.zip › case4/test1/MildDemented/Copy of 28 (12).jpg]

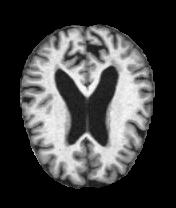

Supplement: Supplemental Information 1 — Image source: https://www.kaggle.com/datasets/tourist55/alzheimers-dataset-4-class-of-images. License: Open Database License (ODbL) v1.0. [file peerj-cs-10-2459-s001.zip › case4/test1/NonDemented/Copy of 26.jpg]

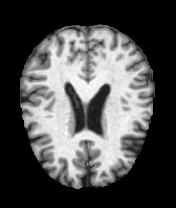

Supplement: Supplemental Information 1 — Image source: https://www.kaggle.com/datasets/tourist55/alzheimers-dataset-4-class-of-images. License: Open Database License (ODbL) v1.0. [file peerj-cs-10-2459-s001.zip › case4/test1/NonDemented/Copy of 26 (93).jpg]

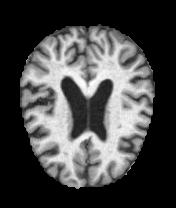

Supplement: Supplemental Information 1 — Image source: https://www.kaggle.com/datasets/tourist55/alzheimers-dataset-4-class-of-images. License: Open Database License (ODbL) v1.0. [file peerj-cs-10-2459-s001.zip › case4/test1/NonDemented/Copy of 27 (21).jpg]

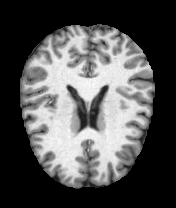

Supplement: Supplemental Information 1 — Image source: https://www.kaggle.com/datasets/tourist55/alzheimers-dataset-4-class-of-images. License: Open Database License (ODbL) v1.0. [file peerj-cs-10-2459-s001.zip › case4/test1/NonDemented/Copy of 26 (79).jpg]

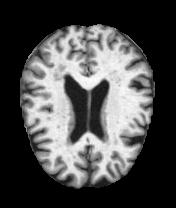

Supplement: Supplemental Information 1 — Image source: https://www.kaggle.com/datasets/tourist55/alzheimers-dataset-4-class-of-images. License: Open Database License (ODbL) v1.0. [file peerj-cs-10-2459-s001.zip › case4/test1/NonDemented/Copy of 26 (99).jpg]

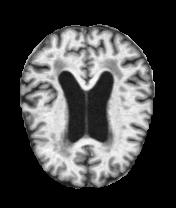

Supplement: Supplemental Information 1 — Image source: https://www.kaggle.com/datasets/tourist55/alzheimers-dataset-4-class-of-images. License: Open Database License (ODbL) v1.0. [file peerj-cs-10-2459-s001.zip › case4/test1/NonDemented/Copy of 26 (94).jpg]

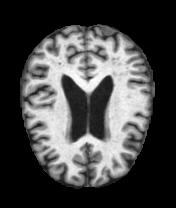

Supplement: Supplemental Information 1 — Image source: https://www.kaggle.com/datasets/tourist55/alzheimers-dataset-4-class-of-images. License: Open Database License (ODbL) v1.0. [file peerj-cs-10-2459-s001.zip › case4/test1/NonDemented/Copy of 26 (82).jpg]

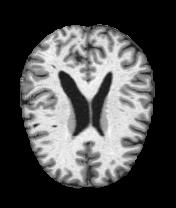

Supplement: Supplemental Information 1 — Image source: https://www.kaggle.com/datasets/tourist55/alzheimers-dataset-4-class-of-images. License: Open Database License (ODbL) v1.0. [file peerj-cs-10-2459-s001.zip › case4/test1/NonDemented/Copy of 26 (88).jpg]

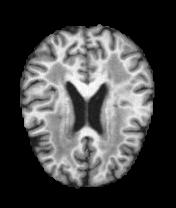

Supplement: Supplemental Information 1 — Image source: https://www.kaggle.com/datasets/tourist55/alzheimers-dataset-4-class-of-images. License: Open Database License (ODbL) v1.0. [file peerj-cs-10-2459-s001.zip › case4/test1/NonDemented/Copy of 27 (10).jpg]

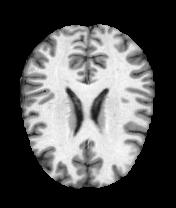

Supplement: Supplemental Information 1 — Image source: https://www.kaggle.com/datasets/tourist55/alzheimers-dataset-4-class-of-images. License: Open Database License (ODbL) v1.0. [file peerj-cs-10-2459-s001.zip › case4/test1/NonDemented/Copy of 26 (70).jpg]

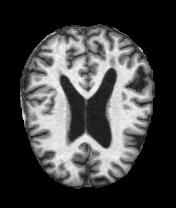

Supplement: Supplemental Information 1 — Image source: https://www.kaggle.com/datasets/tourist55/alzheimers-dataset-4-class-of-images. License: Open Database License (ODbL) v1.0. [file peerj-cs-10-2459-s001.zip › case4/test1/NonDemented/Copy of 26 (89).jpg]

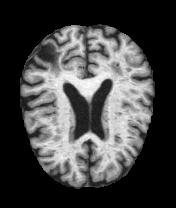

Supplement: Supplemental Information 1 — Image source: https://www.kaggle.com/datasets/tourist55/alzheimers-dataset-4-class-of-images. License: Open Database License (ODbL) v1.0. [file peerj-cs-10-2459-s001.zip › case4/test1/NonDemented/Copy of 26 (68).jpg]

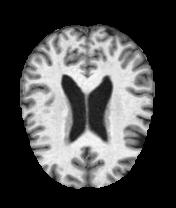

Supplement: Supplemental Information 1 — Image source: https://www.kaggle.com/datasets/tourist55/alzheimers-dataset-4-class-of-images. License: Open Database License (ODbL) v1.0. [file peerj-cs-10-2459-s001.zip › case4/test1/NonDemented/Copy of 26 (91).jpg]

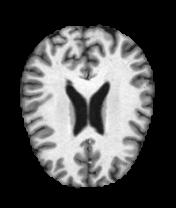

Supplement: Supplemental Information 1 — Image source: https://www.kaggle.com/datasets/tourist55/alzheimers-dataset-4-class-of-images. License: Open Database License (ODbL) v1.0. [file peerj-cs-10-2459-s001.zip › case4/test1/NonDemented/Copy of 27 (12).jpg]

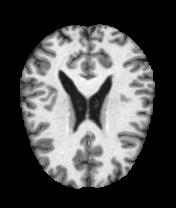

Supplement: Supplemental Information 1 — Image source: https://www.kaggle.com/datasets/tourist55/alzheimers-dataset-4-class-of-images. License: Open Database License (ODbL) v1.0. [file peerj-cs-10-2459-s001.zip › case4/test1/NonDemented/Copy of 26 (96).jpg]

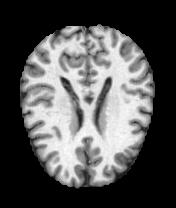

Supplement: Supplemental Information 1 — Image source: https://www.kaggle.com/datasets/tourist55/alzheimers-dataset-4-class-of-images. License: Open Database License (ODbL) v1.0. [file peerj-cs-10-2459-s001.zip › case4/test1/NonDemented/Copy of 27 (2).jpg]

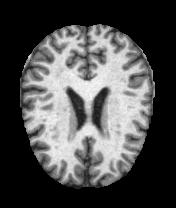

Supplement: Supplemental Information 1 — Image source: https://www.kaggle.com/datasets/tourist55/alzheimers-dataset-4-class-of-images. License: Open Database License (ODbL) v1.0. [file peerj-cs-10-2459-s001.zip › case4/test1/NonDemented/Copy of 26 (85).jpg]

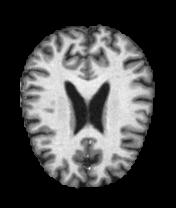

Supplement: Supplemental Information 1 — Image source: https://www.kaggle.com/datasets/tourist55/alzheimers-dataset-4-class-of-images. License: Open Database License (ODbL) v1.0. [file peerj-cs-10-2459-s001.zip › case4/test1/NonDemented/Copy of 27 (100).jpg]

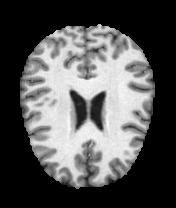

Supplement: Supplemental Information 1 — Image source: https://www.kaggle.com/datasets/tourist55/alzheimers-dataset-4-class-of-images. License: Open Database License (ODbL) v1.0. [file peerj-cs-10-2459-s001.zip › case4/test1/NonDemented/Copy of 27 (19).jpg]

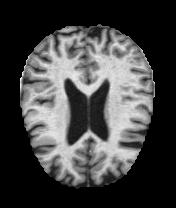

Supplement: Supplemental Information 1 — Image source: https://www.kaggle.com/datasets/tourist55/alzheimers-dataset-4-class-of-images. License: Open Database License (ODbL) v1.0. [file peerj-cs-10-2459-s001.zip › case4/test1/NonDemented/Copy of 27 (33).jpg]
