# Supplementary figures and images for: Enhancing Alzheimer’s disease classification through split federated learning and GANs for imbalanced datasets (part 1 of 4)
Source: PeerJ Comput Sci. 2024 Nov 29;10:e2459. doi: 10.7717/peerj-cs.2459 (PMC11623002; doi:10.7717/peerj-cs.2459)

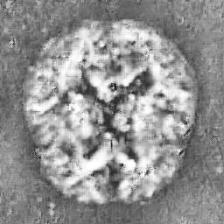

Supplement: Supplemental Information 1 — Image source: https://www.kaggle.com/datasets/tourist55/alzheimers-dataset-4-class-of-images. License: Open Database License (ODbL) v1.0. [file peerj-cs-10-2459-s001.zip › case4/test1/ModerateDemented/Copy of Copy of generated_image_class0_0_31.png]

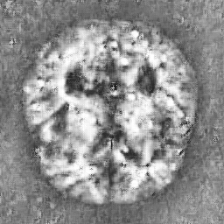

Supplement: Supplemental Information 1 — Image source: https://www.kaggle.com/datasets/tourist55/alzheimers-dataset-4-class-of-images. License: Open Database License (ODbL) v1.0. [file peerj-cs-10-2459-s001.zip › case4/test1/ModerateDemented/Copy of Copy of generated_image_class0_0_48.png]

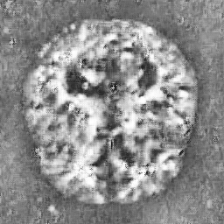

Supplement: Supplemental Information 1 — Image source: https://www.kaggle.com/datasets/tourist55/alzheimers-dataset-4-class-of-images. License: Open Database License (ODbL) v1.0. [file peerj-cs-10-2459-s001.zip › case4/test1/ModerateDemented/Copy of Copy of generated_image_class0_0_50.png]

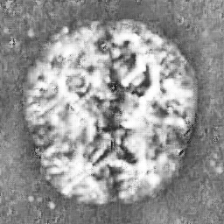

Supplement: Supplemental Information 1 — Image source: https://www.kaggle.com/datasets/tourist55/alzheimers-dataset-4-class-of-images. License: Open Database License (ODbL) v1.0. [file peerj-cs-10-2459-s001.zip › case4/test1/ModerateDemented/Copy of Copy of generated_image_class0_0_42.png]

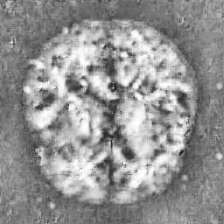

Supplement: Supplemental Information 1 — Image source: https://www.kaggle.com/datasets/tourist55/alzheimers-dataset-4-class-of-images. License: Open Database License (ODbL) v1.0. [file peerj-cs-10-2459-s001.zip › case4/test1/ModerateDemented/Copy of Copy of generated_image_class0_0_43.png]

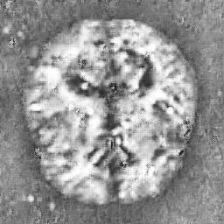

Supplement: Supplemental Information 1 — Image source: https://www.kaggle.com/datasets/tourist55/alzheimers-dataset-4-class-of-images. License: Open Database License (ODbL) v1.0. [file peerj-cs-10-2459-s001.zip › case4/test1/ModerateDemented/Copy of Copy of generated_image_class0_0_49.png]

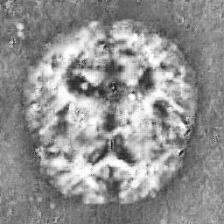

Supplement: Supplemental Information 1 — Image source: https://www.kaggle.com/datasets/tourist55/alzheimers-dataset-4-class-of-images. License: Open Database License (ODbL) v1.0. [file peerj-cs-10-2459-s001.zip › case4/test1/ModerateDemented/Copy of Copy of generated_image_class0_0_41.png]

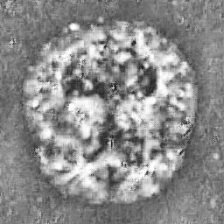

Supplement: Supplemental Information 1 — Image source: https://www.kaggle.com/datasets/tourist55/alzheimers-dataset-4-class-of-images. License: Open Database License (ODbL) v1.0. [file peerj-cs-10-2459-s001.zip › case4/test1/ModerateDemented/Copy of Copy of generated_image_class0_0_47.png]

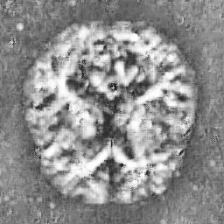

Supplement: Supplemental Information 1 — Image source: https://www.kaggle.com/datasets/tourist55/alzheimers-dataset-4-class-of-images. License: Open Database License (ODbL) v1.0. [file peerj-cs-10-2459-s001.zip › case4/test1/ModerateDemented/Copy of Copy of generated_image_class0_0_46.png]

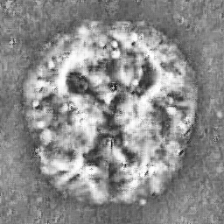

Supplement: Supplemental Information 1 — Image source: https://www.kaggle.com/datasets/tourist55/alzheimers-dataset-4-class-of-images. License: Open Database License (ODbL) v1.0. [file peerj-cs-10-2459-s001.zip › case4/test1/ModerateDemented/Copy of Copy of generated_image_class0_0_45.png]

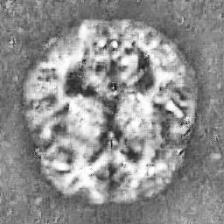

Supplement: Supplemental Information 1 — Image source: https://www.kaggle.com/datasets/tourist55/alzheimers-dataset-4-class-of-images. License: Open Database License (ODbL) v1.0. [file peerj-cs-10-2459-s001.zip › case4/test1/ModerateDemented/Copy of Copy of generated_image_class0_0_38.png]

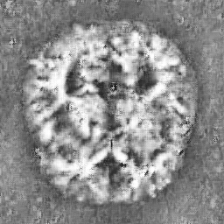

Supplement: Supplemental Information 1 — Image source: https://www.kaggle.com/datasets/tourist55/alzheimers-dataset-4-class-of-images. License: Open Database License (ODbL) v1.0. [file peerj-cs-10-2459-s001.zip › case4/test1/ModerateDemented/Copy of Copy of generated_image_class0_0_24.png]

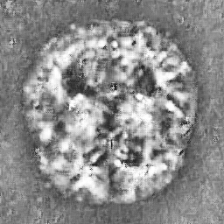

Supplement: Supplemental Information 1 — Image source: https://www.kaggle.com/datasets/tourist55/alzheimers-dataset-4-class-of-images. License: Open Database License (ODbL) v1.0. [file peerj-cs-10-2459-s001.zip › case4/test1/ModerateDemented/Copy of Copy of generated_image_class0_0_40.png]

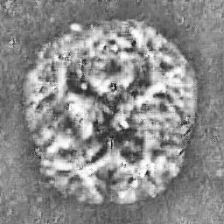

Supplement: Supplemental Information 1 — Image source: https://www.kaggle.com/datasets/tourist55/alzheimers-dataset-4-class-of-images. License: Open Database License (ODbL) v1.0. [file peerj-cs-10-2459-s001.zip › case4/test1/ModerateDemented/Copy of Copy of generated_image_class0_0_33.png]

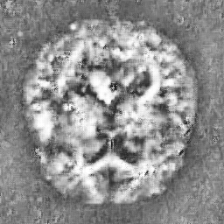

Supplement: Supplemental Information 1 — Image source: https://www.kaggle.com/datasets/tourist55/alzheimers-dataset-4-class-of-images. License: Open Database License (ODbL) v1.0. [file peerj-cs-10-2459-s001.zip › case4/test1/ModerateDemented/Copy of Copy of generated_image_class0_0_37.png]

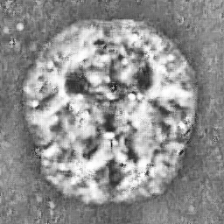

Supplement: Supplemental Information 1 — Image source: https://www.kaggle.com/datasets/tourist55/alzheimers-dataset-4-class-of-images. License: Open Database License (ODbL) v1.0. [file peerj-cs-10-2459-s001.zip › case4/test1/ModerateDemented/Copy of Copy of generated_image_class0_0_44.png]

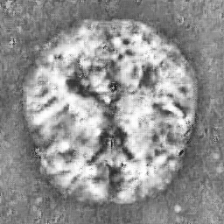

Supplement: Supplemental Information 1 — Image source: https://www.kaggle.com/datasets/tourist55/alzheimers-dataset-4-class-of-images. License: Open Database License (ODbL) v1.0. [file peerj-cs-10-2459-s001.zip › case4/test1/ModerateDemented/Copy of Copy of generated_image_class0_0_25.png]

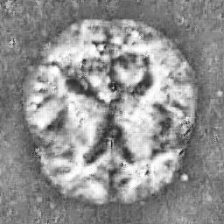

Supplement: Supplemental Information 1 — Image source: https://www.kaggle.com/datasets/tourist55/alzheimers-dataset-4-class-of-images. License: Open Database License (ODbL) v1.0. [file peerj-cs-10-2459-s001.zip › case4/test1/ModerateDemented/Copy of Copy of generated_image_class0_0_34.png]

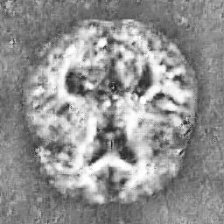

Supplement: Supplemental Information 1 — Image source: https://www.kaggle.com/datasets/tourist55/alzheimers-dataset-4-class-of-images. License: Open Database License (ODbL) v1.0. [file peerj-cs-10-2459-s001.zip › case4/test1/ModerateDemented/Copy of Copy of generated_image_class0_0_30.png]

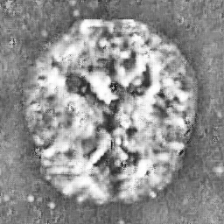

Supplement: Supplemental Information 1 — Image source: https://www.kaggle.com/datasets/tourist55/alzheimers-dataset-4-class-of-images. License: Open Database License (ODbL) v1.0. [file peerj-cs-10-2459-s001.zip › case4/test1/ModerateDemented/Copy of Copy of generated_image_class0_0_11.png]

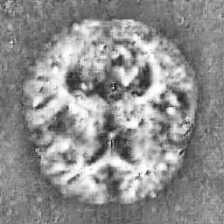

Supplement: Supplemental Information 1 — Image source: https://www.kaggle.com/datasets/tourist55/alzheimers-dataset-4-class-of-images. License: Open Database License (ODbL) v1.0. [file peerj-cs-10-2459-s001.zip › case4/test1/ModerateDemented/Copy of Copy of generated_image_class0_0_39.png]

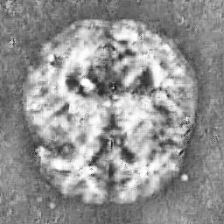

Supplement: Supplemental Information 1 — Image source: https://www.kaggle.com/datasets/tourist55/alzheimers-dataset-4-class-of-images. License: Open Database License (ODbL) v1.0. [file peerj-cs-10-2459-s001.zip › case4/test1/ModerateDemented/Copy of Copy of generated_image_class0_0_23.png]

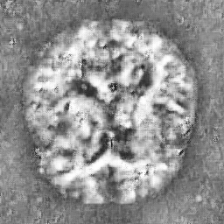

Supplement: Supplemental Information 1 — Image source: https://www.kaggle.com/datasets/tourist55/alzheimers-dataset-4-class-of-images. License: Open Database License (ODbL) v1.0. [file peerj-cs-10-2459-s001.zip › case4/test1/ModerateDemented/Copy of Copy of generated_image_class0_0_27.png]

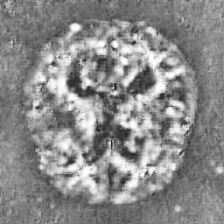

Supplement: Supplemental Information 1 — Image source: https://www.kaggle.com/datasets/tourist55/alzheimers-dataset-4-class-of-images. License: Open Database License (ODbL) v1.0. [file peerj-cs-10-2459-s001.zip › case4/test1/ModerateDemented/Copy of Copy of generated_image_class0_0_20.png]

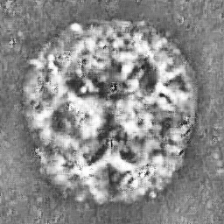

Supplement: Supplemental Information 1 — Image source: https://www.kaggle.com/datasets/tourist55/alzheimers-dataset-4-class-of-images. License: Open Database License (ODbL) v1.0. [file peerj-cs-10-2459-s001.zip › case4/test1/ModerateDemented/Copy of Copy of generated_image_class0_0_28.png]

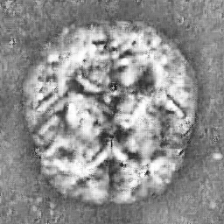

Supplement: Supplemental Information 1 — Image source: https://www.kaggle.com/datasets/tourist55/alzheimers-dataset-4-class-of-images. License: Open Database License (ODbL) v1.0. [file peerj-cs-10-2459-s001.zip › case4/test1/ModerateDemented/Copy of Copy of generated_image_class0_0_36.png]

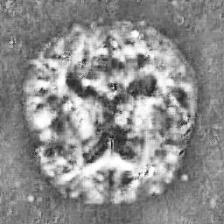

Supplement: Supplemental Information 1 — Image source: https://www.kaggle.com/datasets/tourist55/alzheimers-dataset-4-class-of-images. License: Open Database License (ODbL) v1.0. [file peerj-cs-10-2459-s001.zip › case4/test1/ModerateDemented/Copy of Copy of generated_image_class0_0_6.png]

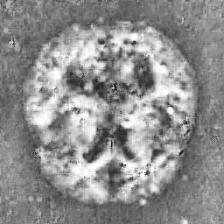

Supplement: Supplemental Information 1 — Image source: https://www.kaggle.com/datasets/tourist55/alzheimers-dataset-4-class-of-images. License: Open Database License (ODbL) v1.0. [file peerj-cs-10-2459-s001.zip › case4/test1/ModerateDemented/Copy of Copy of generated_image_class0_0_26.png]

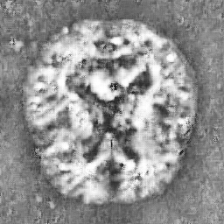

Supplement: Supplemental Information 1 — Image source: https://www.kaggle.com/datasets/tourist55/alzheimers-dataset-4-class-of-images. License: Open Database License (ODbL) v1.0. [file peerj-cs-10-2459-s001.zip › case4/test1/ModerateDemented/Copy of Copy of generated_image_class0_0_29.png]

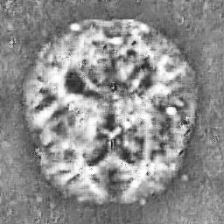

Supplement: Supplemental Information 1 — Image source: https://www.kaggle.com/datasets/tourist55/alzheimers-dataset-4-class-of-images. License: Open Database License (ODbL) v1.0. [file peerj-cs-10-2459-s001.zip › case4/test1/ModerateDemented/Copy of Copy of generated_image_class0_0_19.png]

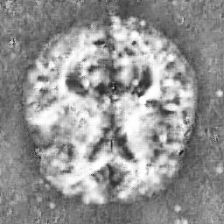

Supplement: Supplemental Information 1 — Image source: https://www.kaggle.com/datasets/tourist55/alzheimers-dataset-4-class-of-images. License: Open Database License (ODbL) v1.0. [file peerj-cs-10-2459-s001.zip › case4/test1/ModerateDemented/Copy of Copy of generated_image_class0_0_22.png]

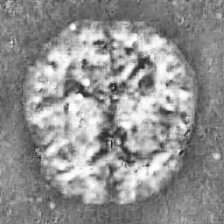

Supplement: Supplemental Information 1 — Image source: https://www.kaggle.com/datasets/tourist55/alzheimers-dataset-4-class-of-images. License: Open Database License (ODbL) v1.0. [file peerj-cs-10-2459-s001.zip › case4/test1/ModerateDemented/Copy of Copy of generated_image_class0_0_12.png]

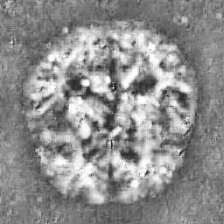

Supplement: Supplemental Information 1 — Image source: https://www.kaggle.com/datasets/tourist55/alzheimers-dataset-4-class-of-images. License: Open Database License (ODbL) v1.0. [file peerj-cs-10-2459-s001.zip › case4/test1/ModerateDemented/Copy of Copy of generated_image_class0_0_35.png]

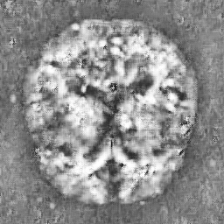

Supplement: Supplemental Information 1 — Image source: https://www.kaggle.com/datasets/tourist55/alzheimers-dataset-4-class-of-images. License: Open Database License (ODbL) v1.0. [file peerj-cs-10-2459-s001.zip › case4/test1/ModerateDemented/Copy of Copy of generated_image_class0_0_32.png]

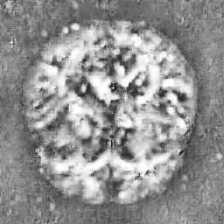

Supplement: Supplemental Information 1 — Image source: https://www.kaggle.com/datasets/tourist55/alzheimers-dataset-4-class-of-images. License: Open Database License (ODbL) v1.0. [file peerj-cs-10-2459-s001.zip › case4/test1/ModerateDemented/Copy of Copy of generated_image_class0_0_5.png]

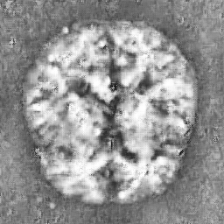

Supplement: Supplemental Information 1 — Image source: https://www.kaggle.com/datasets/tourist55/alzheimers-dataset-4-class-of-images. License: Open Database License (ODbL) v1.0. [file peerj-cs-10-2459-s001.zip › case4/test1/ModerateDemented/Copy of Copy of generated_image_class0_0_14.png]

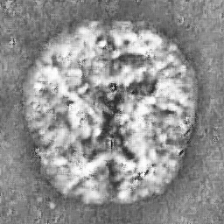

Supplement: Supplemental Information 1 — Image source: https://www.kaggle.com/datasets/tourist55/alzheimers-dataset-4-class-of-images. License: Open Database License (ODbL) v1.0. [file peerj-cs-10-2459-s001.zip › case4/test1/ModerateDemented/Copy of Copy of generated_image_class0_0_18.png]

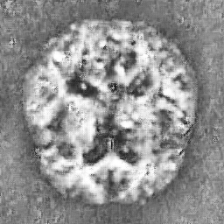

Supplement: Supplemental Information 1 — Image source: https://www.kaggle.com/datasets/tourist55/alzheimers-dataset-4-class-of-images. License: Open Database License (ODbL) v1.0. [file peerj-cs-10-2459-s001.zip › case4/test1/ModerateDemented/Copy of Copy of generated_image_class0_0_8.png]

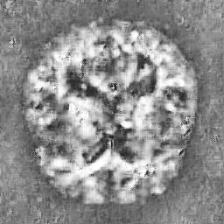

Supplement: Supplemental Information 1 — Image source: https://www.kaggle.com/datasets/tourist55/alzheimers-dataset-4-class-of-images. License: Open Database License (ODbL) v1.0. [file peerj-cs-10-2459-s001.zip › case4/test1/ModerateDemented/Copy of Copy of generated_image_class0_0_15.png]

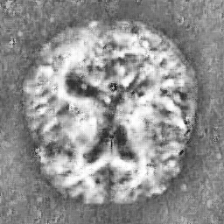

Supplement: Supplemental Information 1 — Image source: https://www.kaggle.com/datasets/tourist55/alzheimers-dataset-4-class-of-images. License: Open Database License (ODbL) v1.0. [file peerj-cs-10-2459-s001.zip › case4/test1/ModerateDemented/Copy of Copy of generated_image_class0_0_21.png]

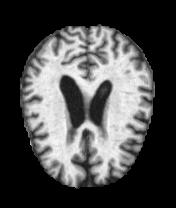

Supplement: Supplemental Information 1 — Image source: https://www.kaggle.com/datasets/tourist55/alzheimers-dataset-4-class-of-images. License: Open Database License (ODbL) v1.0. [file peerj-cs-10-2459-s001.zip › case4/test1/ModerateDemented/Copy of 27.jpg]

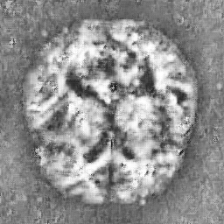

Supplement: Supplemental Information 1 — Image source: https://www.kaggle.com/datasets/tourist55/alzheimers-dataset-4-class-of-images. License: Open Database License (ODbL) v1.0. [file peerj-cs-10-2459-s001.zip › case4/test1/ModerateDemented/Copy of Copy of generated_image_class0_0_7.png]

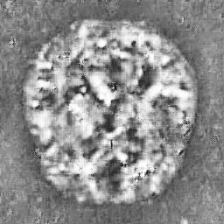

Supplement: Supplemental Information 1 — Image source: https://www.kaggle.com/datasets/tourist55/alzheimers-dataset-4-class-of-images. License: Open Database License (ODbL) v1.0. [file peerj-cs-10-2459-s001.zip › case4/test1/ModerateDemented/Copy of Copy of generated_image_class0_0_2.png]

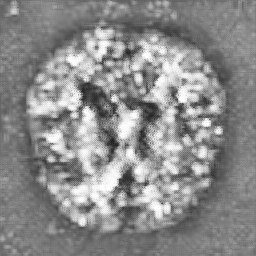

Supplement: Supplemental Information 1 — Image source: https://www.kaggle.com/datasets/tourist55/alzheimers-dataset-4-class-of-images. License: Open Database License (ODbL) v1.0. [file peerj-cs-10-2459-s001.zip › case4/test1/ModerateDemented/generated_image_100.png]

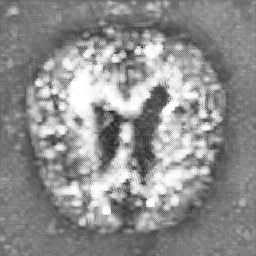

Supplement: Supplemental Information 1 — Image source: https://www.kaggle.com/datasets/tourist55/alzheimers-dataset-4-class-of-images. License: Open Database License (ODbL) v1.0. [file peerj-cs-10-2459-s001.zip › case4/test1/ModerateDemented/generated_image_97.png]

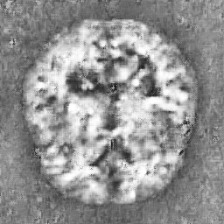

Supplement: Supplemental Information 1 — Image source: https://www.kaggle.com/datasets/tourist55/alzheimers-dataset-4-class-of-images. License: Open Database License (ODbL) v1.0. [file peerj-cs-10-2459-s001.zip › case4/test1/ModerateDemented/Copy of Copy of generated_image_class0_0_3.png]

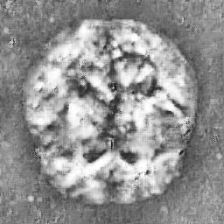

Supplement: Supplemental Information 1 — Image source: https://www.kaggle.com/datasets/tourist55/alzheimers-dataset-4-class-of-images. License: Open Database License (ODbL) v1.0. [file peerj-cs-10-2459-s001.zip › case4/test1/ModerateDemented/Copy of Copy of generated_image_class0_0_13.png]

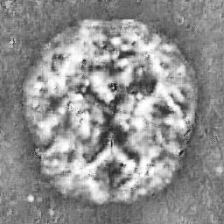

Supplement: Supplemental Information 1 — Image source: https://www.kaggle.com/datasets/tourist55/alzheimers-dataset-4-class-of-images. License: Open Database License (ODbL) v1.0. [file peerj-cs-10-2459-s001.zip › case4/test1/ModerateDemented/Copy of Copy of generated_image_class0_0_4.png]

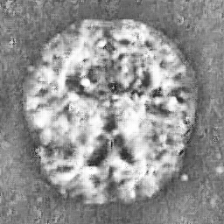

Supplement: Supplemental Information 1 — Image source: https://www.kaggle.com/datasets/tourist55/alzheimers-dataset-4-class-of-images. License: Open Database License (ODbL) v1.0. [file peerj-cs-10-2459-s001.zip › case4/test1/ModerateDemented/Copy of Copy of generated_image_class0_0_17.png]

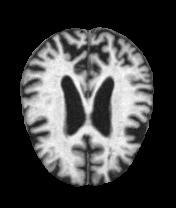

Supplement: Supplemental Information 1 — Image source: https://www.kaggle.com/datasets/tourist55/alzheimers-dataset-4-class-of-images. License: Open Database License (ODbL) v1.0. [file peerj-cs-10-2459-s001.zip › case4/test1/ModerateDemented/Copy of 32 (2).jpg]

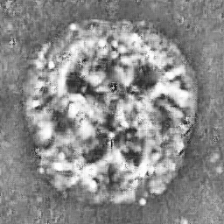

Supplement: Supplemental Information 1 — Image source: https://www.kaggle.com/datasets/tourist55/alzheimers-dataset-4-class-of-images. License: Open Database License (ODbL) v1.0. [file peerj-cs-10-2459-s001.zip › case4/test1/ModerateDemented/Copy of Copy of generated_image_class0_0_16.png]

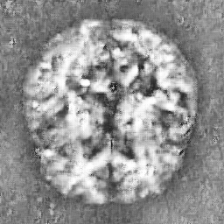

Supplement: Supplemental Information 1 — Image source: https://www.kaggle.com/datasets/tourist55/alzheimers-dataset-4-class-of-images. License: Open Database License (ODbL) v1.0. [file peerj-cs-10-2459-s001.zip › case4/test1/ModerateDemented/Copy of Copy of generated_image_class0_0_1.png]

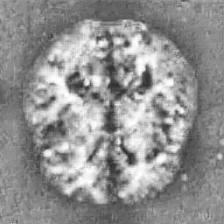

Supplement: Supplemental Information 1 — Image source: https://www.kaggle.com/datasets/tourist55/alzheimers-dataset-4-class-of-images. License: Open Database License (ODbL) v1.0. [file peerj-cs-10-2459-s001.zip › case4/test1/MildDemented/Copy of Copy of generated_image_class_1_53.png]

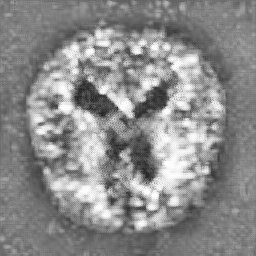

Supplement: Supplemental Information 1 — Image source: https://www.kaggle.com/datasets/tourist55/alzheimers-dataset-4-class-of-images. License: Open Database License (ODbL) v1.0. [file peerj-cs-10-2459-s001.zip › case4/test1/ModerateDemented/generated_image_99.png]

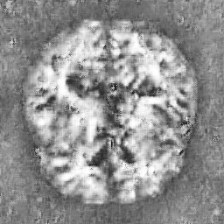

Supplement: Supplemental Information 1 — Image source: https://www.kaggle.com/datasets/tourist55/alzheimers-dataset-4-class-of-images. License: Open Database License (ODbL) v1.0. [file peerj-cs-10-2459-s001.zip › case4/test1/ModerateDemented/Copy of Copy of generated_image_class0_0_10.png]

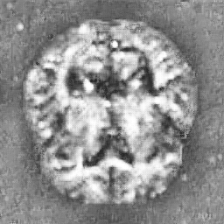

Supplement: Supplemental Information 1 — Image source: https://www.kaggle.com/datasets/tourist55/alzheimers-dataset-4-class-of-images. License: Open Database License (ODbL) v1.0. [file peerj-cs-10-2459-s001.zip › case4/test1/MildDemented/Copy of Copy of generated_image_class_1_58.png]

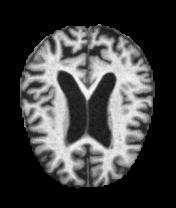

Supplement: Supplemental Information 1 — Image source: https://www.kaggle.com/datasets/tourist55/alzheimers-dataset-4-class-of-images. License: Open Database License (ODbL) v1.0. [file peerj-cs-10-2459-s001.zip › case4/test1/ModerateDemented/Copy of 27 (2).jpg]

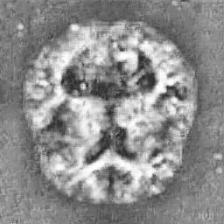

Supplement: Supplemental Information 1 — Image source: https://www.kaggle.com/datasets/tourist55/alzheimers-dataset-4-class-of-images. License: Open Database License (ODbL) v1.0. [file peerj-cs-10-2459-s001.zip › case4/test1/MildDemented/Copy of Copy of generated_image_class_1_46.png]

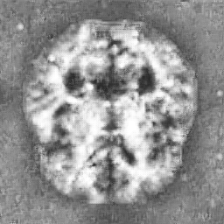

Supplement: Supplemental Information 1 — Image source: https://www.kaggle.com/datasets/tourist55/alzheimers-dataset-4-class-of-images. License: Open Database License (ODbL) v1.0. [file peerj-cs-10-2459-s001.zip › case4/test1/MildDemented/Copy of Copy of generated_image_class_1_52.png]

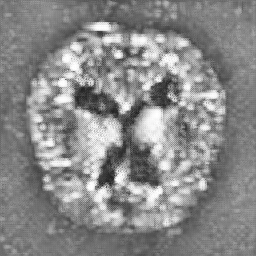

Supplement: Supplemental Information 1 — Image source: https://www.kaggle.com/datasets/tourist55/alzheimers-dataset-4-class-of-images. License: Open Database License (ODbL) v1.0. [file peerj-cs-10-2459-s001.zip › case4/test1/ModerateDemented/generated_image_98.png]

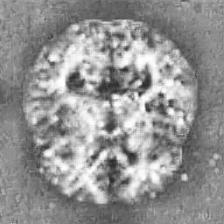

Supplement: Supplemental Information 1 — Image source: https://www.kaggle.com/datasets/tourist55/alzheimers-dataset-4-class-of-images. License: Open Database License (ODbL) v1.0. [file peerj-cs-10-2459-s001.zip › case4/test1/MildDemented/Copy of Copy of generated_image_class_1_59.png]

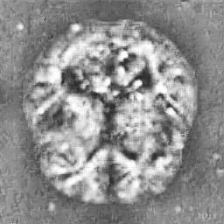

Supplement: Supplemental Information 1 — Image source: https://www.kaggle.com/datasets/tourist55/alzheimers-dataset-4-class-of-images. License: Open Database License (ODbL) v1.0. [file peerj-cs-10-2459-s001.zip › case4/test1/MildDemented/Copy of Copy of generated_image_class_1_47.png]

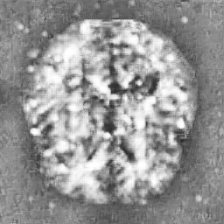

Supplement: Supplemental Information 1 — Image source: https://www.kaggle.com/datasets/tourist55/alzheimers-dataset-4-class-of-images. License: Open Database License (ODbL) v1.0. [file peerj-cs-10-2459-s001.zip › case4/test1/MildDemented/Copy of Copy of generated_image_class_1_45.png]

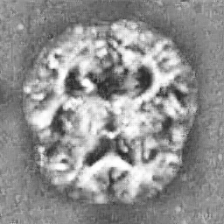

Supplement: Supplemental Information 1 — Image source: https://www.kaggle.com/datasets/tourist55/alzheimers-dataset-4-class-of-images. License: Open Database License (ODbL) v1.0. [file peerj-cs-10-2459-s001.zip › case4/test1/MildDemented/Copy of Copy of generated_image_class_1_42.png]

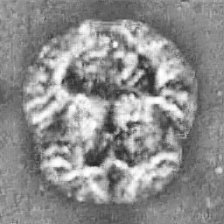

Supplement: Supplemental Information 1 — Image source: https://www.kaggle.com/datasets/tourist55/alzheimers-dataset-4-class-of-images. License: Open Database License (ODbL) v1.0. [file peerj-cs-10-2459-s001.zip › case4/test1/MildDemented/Copy of Copy of generated_image_class_1_50.png]

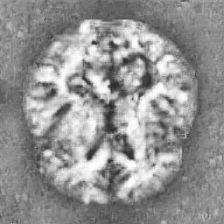

Supplement: Supplemental Information 1 — Image source: https://www.kaggle.com/datasets/tourist55/alzheimers-dataset-4-class-of-images. License: Open Database License (ODbL) v1.0. [file peerj-cs-10-2459-s001.zip › case4/test1/MildDemented/Copy of Copy of generated_image_class_1_49.png]

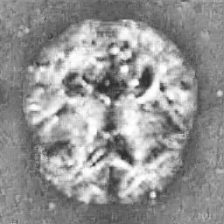

Supplement: Supplemental Information 1 — Image source: https://www.kaggle.com/datasets/tourist55/alzheimers-dataset-4-class-of-images. License: Open Database License (ODbL) v1.0. [file peerj-cs-10-2459-s001.zip › case4/test1/MildDemented/Copy of Copy of generated_image_class_1_33.png]

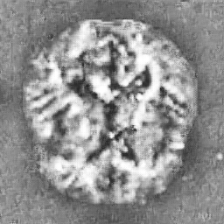

Supplement: Supplemental Information 1 — Image source: https://www.kaggle.com/datasets/tourist55/alzheimers-dataset-4-class-of-images. License: Open Database License (ODbL) v1.0. [file peerj-cs-10-2459-s001.zip › case4/test1/MildDemented/Copy of Copy of generated_image_class_1_41.png]

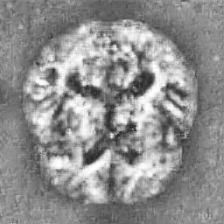

Supplement: Supplemental Information 1 — Image source: https://www.kaggle.com/datasets/tourist55/alzheimers-dataset-4-class-of-images. License: Open Database License (ODbL) v1.0. [file peerj-cs-10-2459-s001.zip › case4/test1/MildDemented/Copy of Copy of generated_image_class_1_40.png]

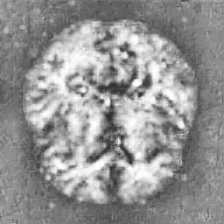

Supplement: Supplemental Information 1 — Image source: https://www.kaggle.com/datasets/tourist55/alzheimers-dataset-4-class-of-images. License: Open Database License (ODbL) v1.0. [file peerj-cs-10-2459-s001.zip › case4/test1/MildDemented/Copy of Copy of generated_image_class_1_56.png]

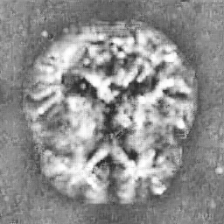

Supplement: Supplemental Information 1 — Image source: https://www.kaggle.com/datasets/tourist55/alzheimers-dataset-4-class-of-images. License: Open Database License (ODbL) v1.0. [file peerj-cs-10-2459-s001.zip › case4/test1/MildDemented/Copy of Copy of generated_image_class_1_48.png]

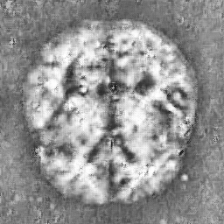

Supplement: Supplemental Information 1 — Image source: https://www.kaggle.com/datasets/tourist55/alzheimers-dataset-4-class-of-images. License: Open Database License (ODbL) v1.0. [file peerj-cs-10-2459-s001.zip › case4/test1/ModerateDemented/Copy of Copy of generated_image_class0_0_9.png]

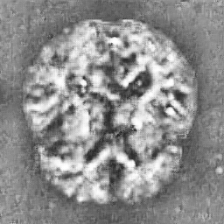

Supplement: Supplemental Information 1 — Image source: https://www.kaggle.com/datasets/tourist55/alzheimers-dataset-4-class-of-images. License: Open Database License (ODbL) v1.0. [file peerj-cs-10-2459-s001.zip › case4/test1/MildDemented/Copy of Copy of generated_image_class_1_34.png]

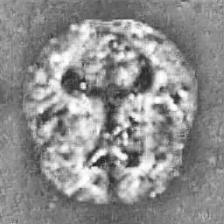

Supplement: Supplemental Information 1 — Image source: https://www.kaggle.com/datasets/tourist55/alzheimers-dataset-4-class-of-images. License: Open Database License (ODbL) v1.0. [file peerj-cs-10-2459-s001.zip › case4/test1/MildDemented/Copy of Copy of generated_image_class_1_37.png]

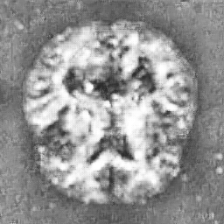

Supplement: Supplemental Information 1 — Image source: https://www.kaggle.com/datasets/tourist55/alzheimers-dataset-4-class-of-images. License: Open Database License (ODbL) v1.0. [file peerj-cs-10-2459-s001.zip › case4/test1/MildDemented/Copy of Copy of generated_image_class_1_36.png]

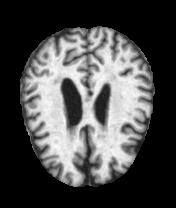

Supplement: Supplemental Information 1 — Image source: https://www.kaggle.com/datasets/tourist55/alzheimers-dataset-4-class-of-images. License: Open Database License (ODbL) v1.0. [file peerj-cs-10-2459-s001.zip › case4/test1/ModerateDemented/Copy of 30.jpg]

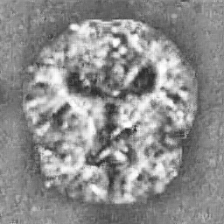

Supplement: Supplemental Information 1 — Image source: https://www.kaggle.com/datasets/tourist55/alzheimers-dataset-4-class-of-images. License: Open Database License (ODbL) v1.0. [file peerj-cs-10-2459-s001.zip › case4/test1/MildDemented/Copy of Copy of generated_image_class_1_39.png]

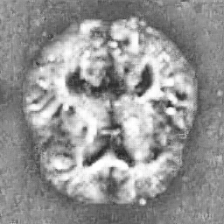

Supplement: Supplemental Information 1 — Image source: https://www.kaggle.com/datasets/tourist55/alzheimers-dataset-4-class-of-images. License: Open Database License (ODbL) v1.0. [file peerj-cs-10-2459-s001.zip › case4/test1/MildDemented/Copy of Copy of generated_image_class_1_38.png]

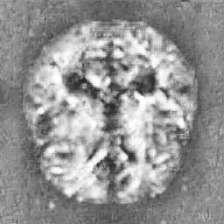

Supplement: Supplemental Information 1 — Image source: https://www.kaggle.com/datasets/tourist55/alzheimers-dataset-4-class-of-images. License: Open Database License (ODbL) v1.0. [file peerj-cs-10-2459-s001.zip › case4/test1/MildDemented/Copy of Copy of generated_image_class_1_26.png]

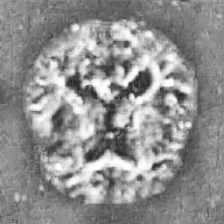

Supplement: Supplemental Information 1 — Image source: https://www.kaggle.com/datasets/tourist55/alzheimers-dataset-4-class-of-images. License: Open Database License (ODbL) v1.0. [file peerj-cs-10-2459-s001.zip › case4/test1/MildDemented/Copy of Copy of generated_image_class_1_22.png]

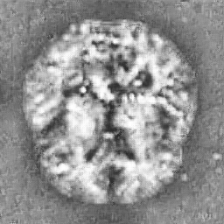

Supplement: Supplemental Information 1 — Image source: https://www.kaggle.com/datasets/tourist55/alzheimers-dataset-4-class-of-images. License: Open Database License (ODbL) v1.0. [file peerj-cs-10-2459-s001.zip › case4/test1/MildDemented/Copy of Copy of generated_image_class_1_35.png]

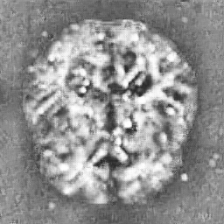

Supplement: Supplemental Information 1 — Image source: https://www.kaggle.com/datasets/tourist55/alzheimers-dataset-4-class-of-images. License: Open Database License (ODbL) v1.0. [file peerj-cs-10-2459-s001.zip › case4/test1/MildDemented/Copy of Copy of generated_image_class_1_43.png]

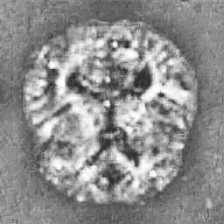

Supplement: Supplemental Information 1 — Image source: https://www.kaggle.com/datasets/tourist55/alzheimers-dataset-4-class-of-images. License: Open Database License (ODbL) v1.0. [file peerj-cs-10-2459-s001.zip › case4/test1/MildDemented/Copy of Copy of generated_image_class_1_24.png]

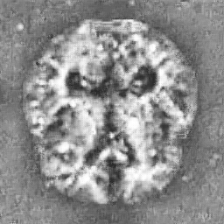

Supplement: Supplemental Information 1 — Image source: https://www.kaggle.com/datasets/tourist55/alzheimers-dataset-4-class-of-images. License: Open Database License (ODbL) v1.0. [file peerj-cs-10-2459-s001.zip › case4/test1/MildDemented/Copy of Copy of generated_image_class_1_29.png]

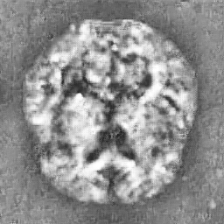

Supplement: Supplemental Information 1 — Image source: https://www.kaggle.com/datasets/tourist55/alzheimers-dataset-4-class-of-images. License: Open Database License (ODbL) v1.0. [file peerj-cs-10-2459-s001.zip › case4/test1/MildDemented/Copy of Copy of generated_image_class_1_25.png]

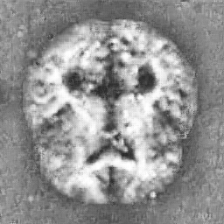

Supplement: Supplemental Information 1 — Image source: https://www.kaggle.com/datasets/tourist55/alzheimers-dataset-4-class-of-images. License: Open Database License (ODbL) v1.0. [file peerj-cs-10-2459-s001.zip › case4/test1/MildDemented/Copy of Copy of generated_image_class_1_21.png]

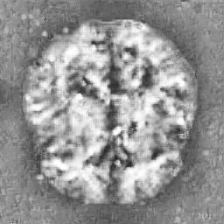

Supplement: Supplemental Information 1 — Image source: https://www.kaggle.com/datasets/tourist55/alzheimers-dataset-4-class-of-images. License: Open Database License (ODbL) v1.0. [file peerj-cs-10-2459-s001.zip › case4/test1/MildDemented/Copy of Copy of generated_image_class_1_44.png]

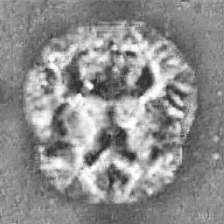

Supplement: Supplemental Information 1 — Image source: https://www.kaggle.com/datasets/tourist55/alzheimers-dataset-4-class-of-images. License: Open Database License (ODbL) v1.0. [file peerj-cs-10-2459-s001.zip › case4/test1/MildDemented/Copy of Copy of generated_image_class_1_30.png]

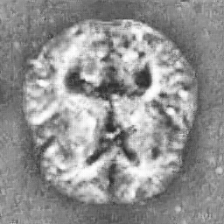

Supplement: Supplemental Information 1 — Image source: https://www.kaggle.com/datasets/tourist55/alzheimers-dataset-4-class-of-images. License: Open Database License (ODbL) v1.0. [file peerj-cs-10-2459-s001.zip › case4/test1/MildDemented/Copy of Copy of generated_image_class_1_23.png]

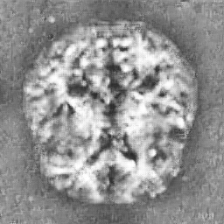

Supplement: Supplemental Information 1 — Image source: https://www.kaggle.com/datasets/tourist55/alzheimers-dataset-4-class-of-images. License: Open Database License (ODbL) v1.0. [file peerj-cs-10-2459-s001.zip › case4/test1/MildDemented/Copy of Copy of generated_image_class_1_19.png]

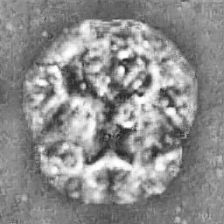

Supplement: Supplemental Information 1 — Image source: https://www.kaggle.com/datasets/tourist55/alzheimers-dataset-4-class-of-images. License: Open Database License (ODbL) v1.0. [file peerj-cs-10-2459-s001.zip › case4/test1/MildDemented/Copy of Copy of generated_image_class_1_14.png]

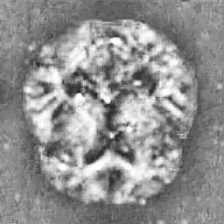

Supplement: Supplemental Information 1 — Image source: https://www.kaggle.com/datasets/tourist55/alzheimers-dataset-4-class-of-images. License: Open Database License (ODbL) v1.0. [file peerj-cs-10-2459-s001.zip › case4/test1/MildDemented/Copy of Copy of generated_image_class_1_20.png]

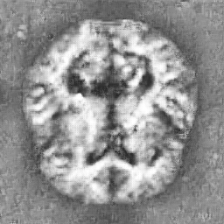

Supplement: Supplemental Information 1 — Image source: https://www.kaggle.com/datasets/tourist55/alzheimers-dataset-4-class-of-images. License: Open Database License (ODbL) v1.0. [file peerj-cs-10-2459-s001.zip › case4/test1/MildDemented/Copy of Copy of generated_image_class_1_16.png]

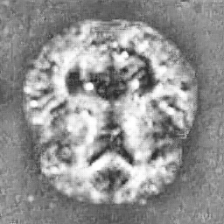

Supplement: Supplemental Information 1 — Image source: https://www.kaggle.com/datasets/tourist55/alzheimers-dataset-4-class-of-images. License: Open Database License (ODbL) v1.0. [file peerj-cs-10-2459-s001.zip › case4/test1/MildDemented/Copy of Copy of generated_image_class_1_31.png]

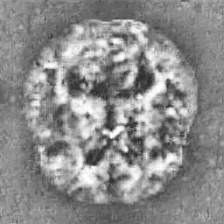

Supplement: Supplemental Information 1 — Image source: https://www.kaggle.com/datasets/tourist55/alzheimers-dataset-4-class-of-images. License: Open Database License (ODbL) v1.0. [file peerj-cs-10-2459-s001.zip › case4/test1/MildDemented/Copy of Copy of generated_image_class_1_13.png]

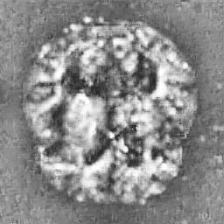

Supplement: Supplemental Information 1 — Image source: https://www.kaggle.com/datasets/tourist55/alzheimers-dataset-4-class-of-images. License: Open Database License (ODbL) v1.0. [file peerj-cs-10-2459-s001.zip › case4/test1/MildDemented/Copy of Copy of generated_image_class_1_11.png]

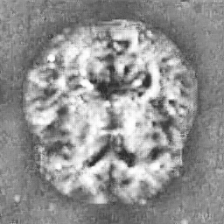

Supplement: Supplemental Information 1 — Image source: https://www.kaggle.com/datasets/tourist55/alzheimers-dataset-4-class-of-images. License: Open Database License (ODbL) v1.0. [file peerj-cs-10-2459-s001.zip › case4/test1/MildDemented/Copy of Copy of generated_image_class_1_15.png]

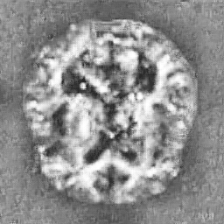

Supplement: Supplemental Information 1 — Image source: https://www.kaggle.com/datasets/tourist55/alzheimers-dataset-4-class-of-images. License: Open Database License (ODbL) v1.0. [file peerj-cs-10-2459-s001.zip › case4/test1/MildDemented/Copy of Copy of generated_image_class_1_18.png]

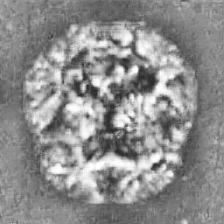

Supplement: Supplemental Information 1 — Image source: https://www.kaggle.com/datasets/tourist55/alzheimers-dataset-4-class-of-images. License: Open Database License (ODbL) v1.0. [file peerj-cs-10-2459-s001.zip › case4/test1/MildDemented/Copy of Copy of generated_image_class_1_4.png]

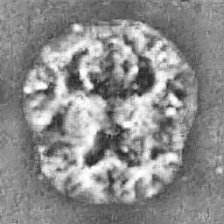

Supplement: Supplemental Information 1 — Image source: https://www.kaggle.com/datasets/tourist55/alzheimers-dataset-4-class-of-images. License: Open Database License (ODbL) v1.0. [file peerj-cs-10-2459-s001.zip › case4/test1/MildDemented/Copy of Copy of generated_image_class_1_3.png]
