# Supplementary figures and images for: Enhancing Alzheimer’s disease classification through split federated learning and GANs for imbalanced datasets (part 4 of 4)
Source: PeerJ Comput Sci. 2024 Nov 29;10:e2459. doi: 10.7717/peerj-cs.2459 (PMC11623002; doi:10.7717/peerj-cs.2459)

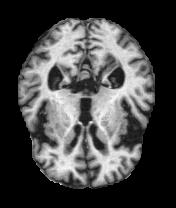

Supplement: Supplemental Information 2 — Image source: https://www.kaggle.com/datasets/tourist55/alzheimers-dataset-4-class-of-images. License: Open Database License (ODbL) v1.0. [file peerj-cs-10-2459-s002.zip › case1_1_4/train/MildDemented/mildDem29.jpg]

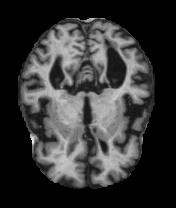

Supplement: Supplemental Information 2 — Image source: https://www.kaggle.com/datasets/tourist55/alzheimers-dataset-4-class-of-images. License: Open Database License (ODbL) v1.0. [file peerj-cs-10-2459-s002.zip › case1_1_4/train/MildDemented/mildDem28.jpg]

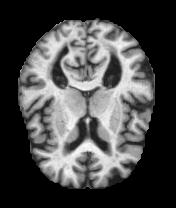

Supplement: Supplemental Information 2 — Image source: https://www.kaggle.com/datasets/tourist55/alzheimers-dataset-4-class-of-images. License: Open Database License (ODbL) v1.0. [file peerj-cs-10-2459-s002.zip › case1_1_4/train/MildDemented/mildDem293.jpg]

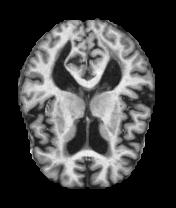

Supplement: Supplemental Information 2 — Image source: https://www.kaggle.com/datasets/tourist55/alzheimers-dataset-4-class-of-images. License: Open Database License (ODbL) v1.0. [file peerj-cs-10-2459-s002.zip › case1_1_4/train/MildDemented/mildDem291.jpg]

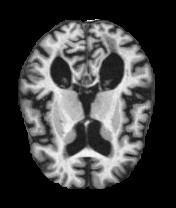

Supplement: Supplemental Information 2 — Image source: https://www.kaggle.com/datasets/tourist55/alzheimers-dataset-4-class-of-images. License: Open Database License (ODbL) v1.0. [file peerj-cs-10-2459-s002.zip › case1_1_4/train/MildDemented/mildDem308.jpg]

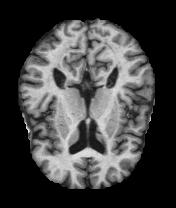

Supplement: Supplemental Information 2 — Image source: https://www.kaggle.com/datasets/tourist55/alzheimers-dataset-4-class-of-images. License: Open Database License (ODbL) v1.0. [file peerj-cs-10-2459-s002.zip › case1_1_4/train/MildDemented/mildDem299.jpg]

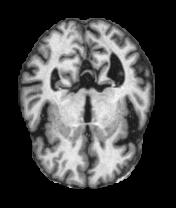

Supplement: Supplemental Information 2 — Image source: https://www.kaggle.com/datasets/tourist55/alzheimers-dataset-4-class-of-images. License: Open Database License (ODbL) v1.0. [file peerj-cs-10-2459-s002.zip › case1_1_4/train/MildDemented/mildDem30.jpg]

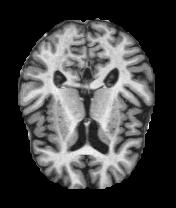

Supplement: Supplemental Information 2 — Image source: https://www.kaggle.com/datasets/tourist55/alzheimers-dataset-4-class-of-images. License: Open Database License (ODbL) v1.0. [file peerj-cs-10-2459-s002.zip › case1_1_4/train/MildDemented/mildDem285.jpg]

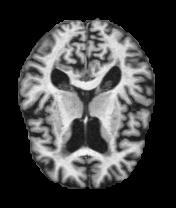

Supplement: Supplemental Information 2 — Image source: https://www.kaggle.com/datasets/tourist55/alzheimers-dataset-4-class-of-images. License: Open Database License (ODbL) v1.0. [file peerj-cs-10-2459-s002.zip › case1_1_4/train/MildDemented/mildDem281.jpg]

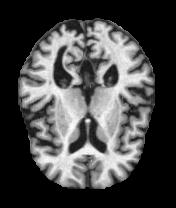

Supplement: Supplemental Information 2 — Image source: https://www.kaggle.com/datasets/tourist55/alzheimers-dataset-4-class-of-images. License: Open Database License (ODbL) v1.0. [file peerj-cs-10-2459-s002.zip › case1_1_4/train/MildDemented/mildDem278.jpg]

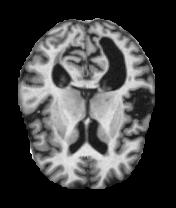

Supplement: Supplemental Information 2 — Image source: https://www.kaggle.com/datasets/tourist55/alzheimers-dataset-4-class-of-images. License: Open Database License (ODbL) v1.0. [file peerj-cs-10-2459-s002.zip › case1_1_4/train/MildDemented/mildDem264.jpg]

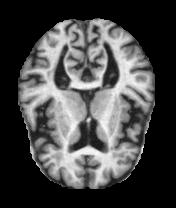

Supplement: Supplemental Information 2 — Image source: https://www.kaggle.com/datasets/tourist55/alzheimers-dataset-4-class-of-images. License: Open Database License (ODbL) v1.0. [file peerj-cs-10-2459-s002.zip › case1_1_4/train/MildDemented/mildDem274.jpg]

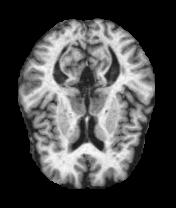

Supplement: Supplemental Information 2 — Image source: https://www.kaggle.com/datasets/tourist55/alzheimers-dataset-4-class-of-images. License: Open Database License (ODbL) v1.0. [file peerj-cs-10-2459-s002.zip › case1_1_4/train/MildDemented/mildDem260.jpg]

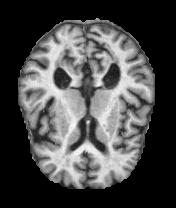

Supplement: Supplemental Information 2 — Image source: https://www.kaggle.com/datasets/tourist55/alzheimers-dataset-4-class-of-images. License: Open Database License (ODbL) v1.0. [file peerj-cs-10-2459-s002.zip › case1_1_4/train/MildDemented/mildDem301.jpg]

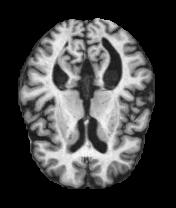

Supplement: Supplemental Information 2 — Image source: https://www.kaggle.com/datasets/tourist55/alzheimers-dataset-4-class-of-images. License: Open Database License (ODbL) v1.0. [file peerj-cs-10-2459-s002.zip › case1_1_4/train/MildDemented/mildDem272.jpg]

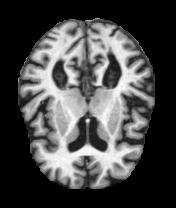

Supplement: Supplemental Information 2 — Image source: https://www.kaggle.com/datasets/tourist55/alzheimers-dataset-4-class-of-images. License: Open Database License (ODbL) v1.0. [file peerj-cs-10-2459-s002.zip › case1_1_4/train/MildDemented/mildDem275.jpg]

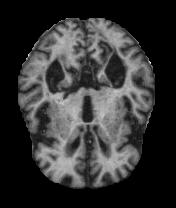

Supplement: Supplemental Information 2 — Image source: https://www.kaggle.com/datasets/tourist55/alzheimers-dataset-4-class-of-images. License: Open Database License (ODbL) v1.0. [file peerj-cs-10-2459-s002.zip › case1_1_4/train/MildDemented/mildDem32.jpg]

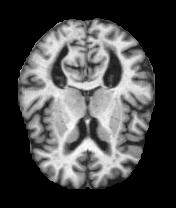

Supplement: Supplemental Information 2 — Image source: https://www.kaggle.com/datasets/tourist55/alzheimers-dataset-4-class-of-images. License: Open Database License (ODbL) v1.0. [file peerj-cs-10-2459-s002.zip › case1_1_4/train/MildDemented/mildDem265.jpg]

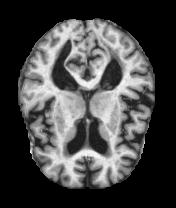

Supplement: Supplemental Information 2 — Image source: https://www.kaggle.com/datasets/tourist55/alzheimers-dataset-4-class-of-images. License: Open Database License (ODbL) v1.0. [file peerj-cs-10-2459-s002.zip › case1_1_4/train/MildDemented/mildDem263.jpg]

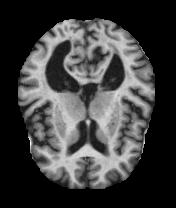

Supplement: Supplemental Information 2 — Image source: https://www.kaggle.com/datasets/tourist55/alzheimers-dataset-4-class-of-images. License: Open Database License (ODbL) v1.0. [file peerj-cs-10-2459-s002.zip › case1_1_4/train/MildDemented/mildDem269.jpg]

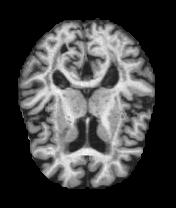

Supplement: Supplemental Information 2 — Image source: https://www.kaggle.com/datasets/tourist55/alzheimers-dataset-4-class-of-images. License: Open Database License (ODbL) v1.0. [file peerj-cs-10-2459-s002.zip › case1_1_4/train/MildDemented/mildDem287.jpg]

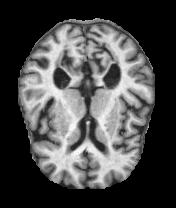

Supplement: Supplemental Information 2 — Image source: https://www.kaggle.com/datasets/tourist55/alzheimers-dataset-4-class-of-images. License: Open Database License (ODbL) v1.0. [file peerj-cs-10-2459-s002.zip › case1_1_4/train/MildDemented/mildDem273.jpg]

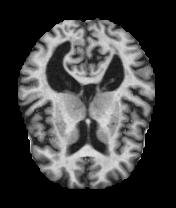

Supplement: Supplemental Information 2 — Image source: https://www.kaggle.com/datasets/tourist55/alzheimers-dataset-4-class-of-images. License: Open Database License (ODbL) v1.0. [file peerj-cs-10-2459-s002.zip › case1_1_4/train/MildDemented/mildDem297.jpg]

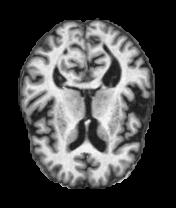

Supplement: Supplemental Information 2 — Image source: https://www.kaggle.com/datasets/tourist55/alzheimers-dataset-4-class-of-images. License: Open Database License (ODbL) v1.0. [file peerj-cs-10-2459-s002.zip › case1_1_4/train/MildDemented/mildDem282.jpg]

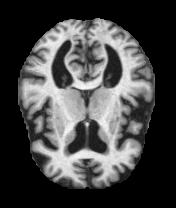

Supplement: Supplemental Information 2 — Image source: https://www.kaggle.com/datasets/tourist55/alzheimers-dataset-4-class-of-images. License: Open Database License (ODbL) v1.0. [file peerj-cs-10-2459-s002.zip › case1_1_4/train/MildDemented/mildDem261.jpg]

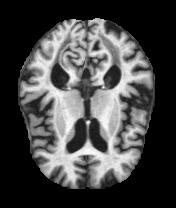

Supplement: Supplemental Information 2 — Image source: https://www.kaggle.com/datasets/tourist55/alzheimers-dataset-4-class-of-images. License: Open Database License (ODbL) v1.0. [file peerj-cs-10-2459-s002.zip › case1_1_4/train/MildDemented/mildDem268.jpg]

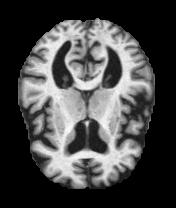

Supplement: Supplemental Information 2 — Image source: https://www.kaggle.com/datasets/tourist55/alzheimers-dataset-4-class-of-images. License: Open Database License (ODbL) v1.0. [file peerj-cs-10-2459-s002.zip › case1_1_4/train/MildDemented/mildDem289.jpg]

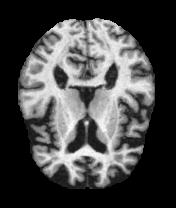

Supplement: Supplemental Information 2 — Image source: https://www.kaggle.com/datasets/tourist55/alzheimers-dataset-4-class-of-images. License: Open Database License (ODbL) v1.0. [file peerj-cs-10-2459-s002.zip › case1_1_4/train/MildDemented/mildDem276.jpg]

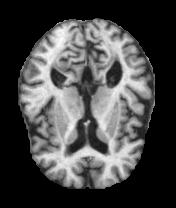

Supplement: Supplemental Information 2 — Image source: https://www.kaggle.com/datasets/tourist55/alzheimers-dataset-4-class-of-images. License: Open Database License (ODbL) v1.0. [file peerj-cs-10-2459-s002.zip › case1_1_4/train/MildDemented/mildDem279.jpg]

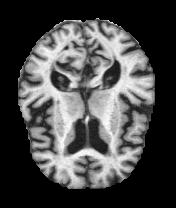

Supplement: Supplemental Information 2 — Image source: https://www.kaggle.com/datasets/tourist55/alzheimers-dataset-4-class-of-images. License: Open Database License (ODbL) v1.0. [file peerj-cs-10-2459-s002.zip › case1_1_4/train/MildDemented/mildDem294.jpg]

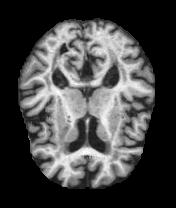

Supplement: Supplemental Information 2 — Image source: https://www.kaggle.com/datasets/tourist55/alzheimers-dataset-4-class-of-images. License: Open Database License (ODbL) v1.0. [file peerj-cs-10-2459-s002.zip › case1_1_4/train/MildDemented/mildDem259.jpg]

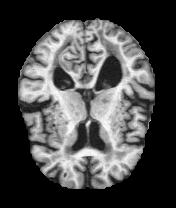

Supplement: Supplemental Information 2 — Image source: https://www.kaggle.com/datasets/tourist55/alzheimers-dataset-4-class-of-images. License: Open Database License (ODbL) v1.0. [file peerj-cs-10-2459-s002.zip › case1_1_4/train/MildDemented/mildDem295.jpg]

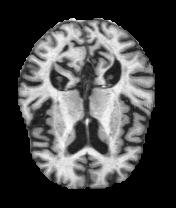

Supplement: Supplemental Information 2 — Image source: https://www.kaggle.com/datasets/tourist55/alzheimers-dataset-4-class-of-images. License: Open Database License (ODbL) v1.0. [file peerj-cs-10-2459-s002.zip › case1_1_4/train/MildDemented/mildDem238.jpg]

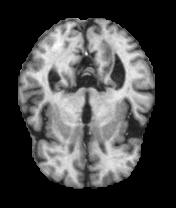

Supplement: Supplemental Information 2 — Image source: https://www.kaggle.com/datasets/tourist55/alzheimers-dataset-4-class-of-images. License: Open Database License (ODbL) v1.0. [file peerj-cs-10-2459-s002.zip › case1_1_4/train/MildDemented/mildDem27.jpg]

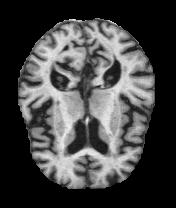

Supplement: Supplemental Information 2 — Image source: https://www.kaggle.com/datasets/tourist55/alzheimers-dataset-4-class-of-images. License: Open Database License (ODbL) v1.0. [file peerj-cs-10-2459-s002.zip › case1_1_4/train/MildDemented/mildDem266.jpg]

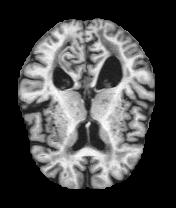

Supplement: Supplemental Information 2 — Image source: https://www.kaggle.com/datasets/tourist55/alzheimers-dataset-4-class-of-images. License: Open Database License (ODbL) v1.0. [file peerj-cs-10-2459-s002.zip › case1_1_4/train/MildDemented/mildDem267.jpg]

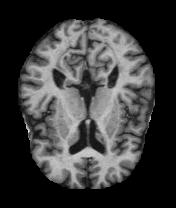

Supplement: Supplemental Information 2 — Image source: https://www.kaggle.com/datasets/tourist55/alzheimers-dataset-4-class-of-images. License: Open Database License (ODbL) v1.0. [file peerj-cs-10-2459-s002.zip › case1_1_4/train/MildDemented/mildDem271.jpg]

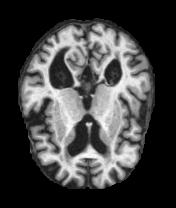

Supplement: Supplemental Information 2 — Image source: https://www.kaggle.com/datasets/tourist55/alzheimers-dataset-4-class-of-images. License: Open Database License (ODbL) v1.0. [file peerj-cs-10-2459-s002.zip › case1_1_4/train/MildDemented/mildDem277.jpg]

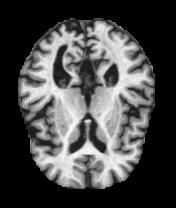

Supplement: Supplemental Information 2 — Image source: https://www.kaggle.com/datasets/tourist55/alzheimers-dataset-4-class-of-images. License: Open Database License (ODbL) v1.0. [file peerj-cs-10-2459-s002.zip › case1_1_4/train/MildDemented/mildDem250.jpg]

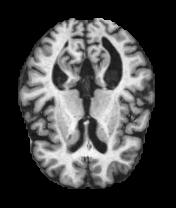

Supplement: Supplemental Information 2 — Image source: https://www.kaggle.com/datasets/tourist55/alzheimers-dataset-4-class-of-images. License: Open Database License (ODbL) v1.0. [file peerj-cs-10-2459-s002.zip › case1_1_4/train/MildDemented/mildDem244.jpg]

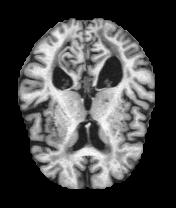

Supplement: Supplemental Information 2 — Image source: https://www.kaggle.com/datasets/tourist55/alzheimers-dataset-4-class-of-images. License: Open Database License (ODbL) v1.0. [file peerj-cs-10-2459-s002.zip › case1_1_4/train/MildDemented/mildDem239.jpg]

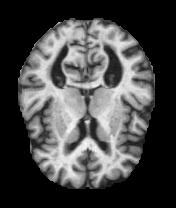

Supplement: Supplemental Information 2 — Image source: https://www.kaggle.com/datasets/tourist55/alzheimers-dataset-4-class-of-images. License: Open Database License (ODbL) v1.0. [file peerj-cs-10-2459-s002.zip › case1_1_4/train/MildDemented/mildDem237.jpg]

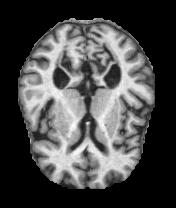

Supplement: Supplemental Information 2 — Image source: https://www.kaggle.com/datasets/tourist55/alzheimers-dataset-4-class-of-images. License: Open Database License (ODbL) v1.0. [file peerj-cs-10-2459-s002.zip › case1_1_4/train/MildDemented/mildDem245.jpg]

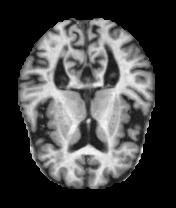

Supplement: Supplemental Information 2 — Image source: https://www.kaggle.com/datasets/tourist55/alzheimers-dataset-4-class-of-images. License: Open Database License (ODbL) v1.0. [file peerj-cs-10-2459-s002.zip › case1_1_4/train/MildDemented/mildDem246.jpg]

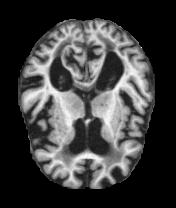

Supplement: Supplemental Information 2 — Image source: https://www.kaggle.com/datasets/tourist55/alzheimers-dataset-4-class-of-images. License: Open Database License (ODbL) v1.0. [file peerj-cs-10-2459-s002.zip › case1_1_4/train/MildDemented/mildDem262.jpg]

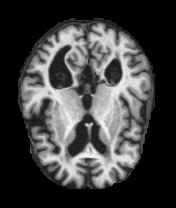

Supplement: Supplemental Information 2 — Image source: https://www.kaggle.com/datasets/tourist55/alzheimers-dataset-4-class-of-images. License: Open Database License (ODbL) v1.0. [file peerj-cs-10-2459-s002.zip › case1_1_4/train/MildDemented/mildDem249.jpg]

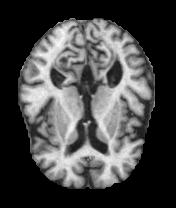

Supplement: Supplemental Information 2 — Image source: https://www.kaggle.com/datasets/tourist55/alzheimers-dataset-4-class-of-images. License: Open Database License (ODbL) v1.0. [file peerj-cs-10-2459-s002.zip › case1_1_4/train/MildDemented/mildDem251.jpg]

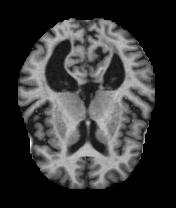

Supplement: Supplemental Information 2 — Image source: https://www.kaggle.com/datasets/tourist55/alzheimers-dataset-4-class-of-images. License: Open Database License (ODbL) v1.0. [file peerj-cs-10-2459-s002.zip › case1_1_4/train/MildDemented/mildDem241.jpg]

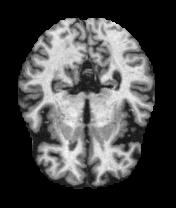

Supplement: Supplemental Information 2 — Image source: https://www.kaggle.com/datasets/tourist55/alzheimers-dataset-4-class-of-images. License: Open Database License (ODbL) v1.0. [file peerj-cs-10-2459-s002.zip › case1_1_4/train/MildDemented/mildDem24.jpg]

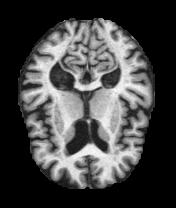

Supplement: Supplemental Information 2 — Image source: https://www.kaggle.com/datasets/tourist55/alzheimers-dataset-4-class-of-images. License: Open Database License (ODbL) v1.0. [file peerj-cs-10-2459-s002.zip › case1_1_4/train/MildDemented/mildDem242.jpg]

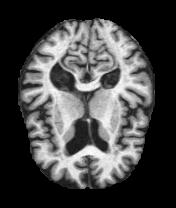

Supplement: Supplemental Information 2 — Image source: https://www.kaggle.com/datasets/tourist55/alzheimers-dataset-4-class-of-images. License: Open Database License (ODbL) v1.0. [file peerj-cs-10-2459-s002.zip › case1_1_4/train/MildDemented/mildDem270.jpg]

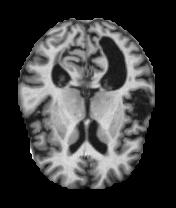

Supplement: Supplemental Information 2 — Image source: https://www.kaggle.com/datasets/tourist55/alzheimers-dataset-4-class-of-images. License: Open Database License (ODbL) v1.0. [file peerj-cs-10-2459-s002.zip › case1_1_4/train/MildDemented/mildDem236.jpg]

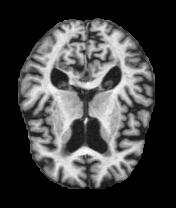

Supplement: Supplemental Information 2 — Image source: https://www.kaggle.com/datasets/tourist55/alzheimers-dataset-4-class-of-images. License: Open Database License (ODbL) v1.0. [file peerj-cs-10-2459-s002.zip › case1_1_4/train/MildDemented/mildDem253.jpg]

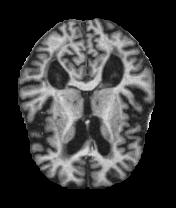

Supplement: Supplemental Information 2 — Image source: https://www.kaggle.com/datasets/tourist55/alzheimers-dataset-4-class-of-images. License: Open Database License (ODbL) v1.0. [file peerj-cs-10-2459-s002.zip › case1_1_4/train/MildDemented/mildDem256.jpg]

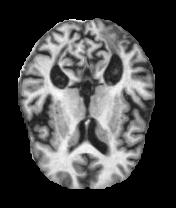

Supplement: Supplemental Information 2 — Image source: https://www.kaggle.com/datasets/tourist55/alzheimers-dataset-4-class-of-images. License: Open Database License (ODbL) v1.0. [file peerj-cs-10-2459-s002.zip › case1_1_4/train/MildDemented/mildDem255.jpg]

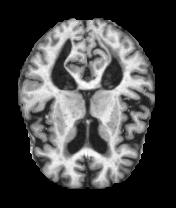

Supplement: Supplemental Information 2 — Image source: https://www.kaggle.com/datasets/tourist55/alzheimers-dataset-4-class-of-images. License: Open Database License (ODbL) v1.0. [file peerj-cs-10-2459-s002.zip › case1_1_4/train/MildDemented/mildDem235.jpg]

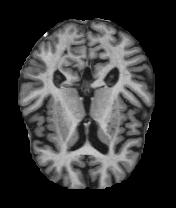

Supplement: Supplemental Information 2 — Image source: https://www.kaggle.com/datasets/tourist55/alzheimers-dataset-4-class-of-images. License: Open Database License (ODbL) v1.0. [file peerj-cs-10-2459-s002.zip › case1_1_4/train/MildDemented/mildDem257.jpg]

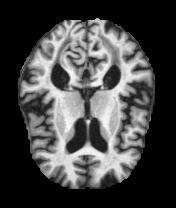

Supplement: Supplemental Information 2 — Image source: https://www.kaggle.com/datasets/tourist55/alzheimers-dataset-4-class-of-images. License: Open Database License (ODbL) v1.0. [file peerj-cs-10-2459-s002.zip › case1_1_4/train/MildDemented/mildDem296.jpg]

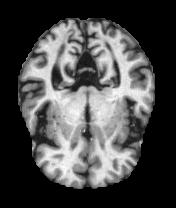

Supplement: Supplemental Information 2 — Image source: https://www.kaggle.com/datasets/tourist55/alzheimers-dataset-4-class-of-images. License: Open Database License (ODbL) v1.0. [file peerj-cs-10-2459-s002.zip › case1_1_4/train/MildDemented/mildDem26.jpg]

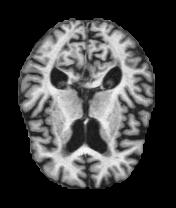

Supplement: Supplemental Information 2 — Image source: https://www.kaggle.com/datasets/tourist55/alzheimers-dataset-4-class-of-images. License: Open Database License (ODbL) v1.0. [file peerj-cs-10-2459-s002.zip › case1_1_4/train/MildDemented/mildDem225.jpg]

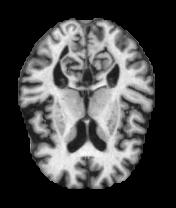

Supplement: Supplemental Information 2 — Image source: https://www.kaggle.com/datasets/tourist55/alzheimers-dataset-4-class-of-images. License: Open Database License (ODbL) v1.0. [file peerj-cs-10-2459-s002.zip › case1_1_4/train/MildDemented/mildDem230.jpg]

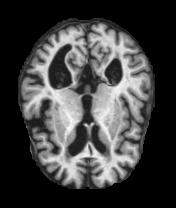

Supplement: Supplemental Information 2 — Image source: https://www.kaggle.com/datasets/tourist55/alzheimers-dataset-4-class-of-images. License: Open Database License (ODbL) v1.0. [file peerj-cs-10-2459-s002.zip › case1_1_4/train/MildDemented/mildDem221.jpg]

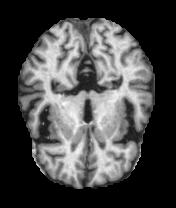

Supplement: Supplemental Information 2 — Image source: https://www.kaggle.com/datasets/tourist55/alzheimers-dataset-4-class-of-images. License: Open Database License (ODbL) v1.0. [file peerj-cs-10-2459-s002.zip › case1_1_4/train/MildDemented/mildDem22.jpg]

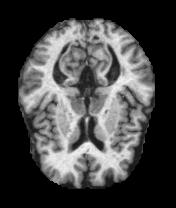

Supplement: Supplemental Information 2 — Image source: https://www.kaggle.com/datasets/tourist55/alzheimers-dataset-4-class-of-images. License: Open Database License (ODbL) v1.0. [file peerj-cs-10-2459-s002.zip › case1_1_4/train/MildDemented/mildDem232.jpg]

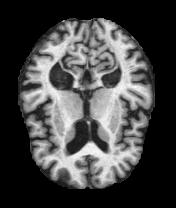

Supplement: Supplemental Information 2 — Image source: https://www.kaggle.com/datasets/tourist55/alzheimers-dataset-4-class-of-images. License: Open Database License (ODbL) v1.0. [file peerj-cs-10-2459-s002.zip › case1_1_4/train/MildDemented/mildDem214.jpg]

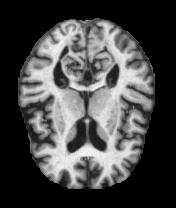

Supplement: Supplemental Information 2 — Image source: https://www.kaggle.com/datasets/tourist55/alzheimers-dataset-4-class-of-images. License: Open Database License (ODbL) v1.0. [file peerj-cs-10-2459-s002.zip › case1_1_4/train/MildDemented/mildDem258.jpg]
