# Supplementary figures and images for: Enhancing Alzheimer’s disease classification through split federated learning and GANs for imbalanced datasets (part 3 of 4)
Source: PeerJ Comput Sci. 2024 Nov 29;10:e2459. doi: 10.7717/peerj-cs.2459 (PMC11623002; doi:10.7717/peerj-cs.2459)

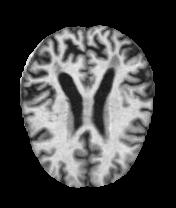

Supplement: Supplemental Information 2 — Image source: https://www.kaggle.com/datasets/tourist55/alzheimers-dataset-4-class-of-images. License: Open Database License (ODbL) v1.0. [file peerj-cs-10-2459-s002.zip › case1_1_4/test/MildDemented/28 (13).jpg]

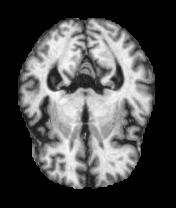

Supplement: Supplemental Information 2 — Image source: https://www.kaggle.com/datasets/tourist55/alzheimers-dataset-4-class-of-images. License: Open Database License (ODbL) v1.0. [file peerj-cs-10-2459-s002.zip › case1_1_4/train/ModerateDemented/moderateDem1.jpg]

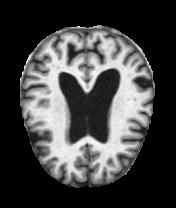

Supplement: Supplemental Information 2 — Image source: https://www.kaggle.com/datasets/tourist55/alzheimers-dataset-4-class-of-images. License: Open Database License (ODbL) v1.0. [file peerj-cs-10-2459-s002.zip › case1_1_4/test/MildDemented/28 (21).jpg]

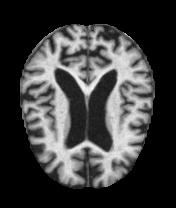

Supplement: Supplemental Information 2 — Image source: https://www.kaggle.com/datasets/tourist55/alzheimers-dataset-4-class-of-images. License: Open Database License (ODbL) v1.0. [file peerj-cs-10-2459-s002.zip › case1_1_4/train/ModerateDemented/moderateDem46.jpg]

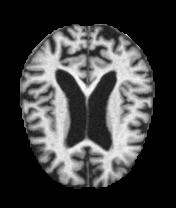

Supplement: Supplemental Information 2 — Image source: https://www.kaggle.com/datasets/tourist55/alzheimers-dataset-4-class-of-images. License: Open Database License (ODbL) v1.0. [file peerj-cs-10-2459-s002.zip › case1_1_4/train/ModerateDemented/moderateDem48.jpg]

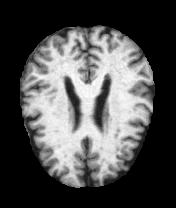

Supplement: Supplemental Information 2 — Image source: https://www.kaggle.com/datasets/tourist55/alzheimers-dataset-4-class-of-images. License: Open Database License (ODbL) v1.0. [file peerj-cs-10-2459-s002.zip › case1_1_4/test/MildDemented/28 (18).jpg]

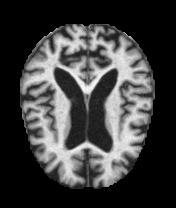

Supplement: Supplemental Information 2 — Image source: https://www.kaggle.com/datasets/tourist55/alzheimers-dataset-4-class-of-images. License: Open Database License (ODbL) v1.0. [file peerj-cs-10-2459-s002.zip › case1_1_4/train/ModerateDemented/moderateDem44.jpg]

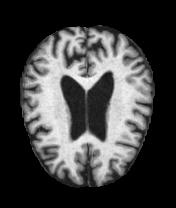

Supplement: Supplemental Information 2 — Image source: https://www.kaggle.com/datasets/tourist55/alzheimers-dataset-4-class-of-images. License: Open Database License (ODbL) v1.0. [file peerj-cs-10-2459-s002.zip › case1_1_4/test/MildDemented/28 (3).jpg]

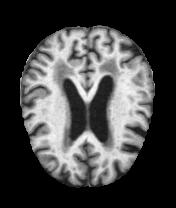

Supplement: Supplemental Information 2 — Image source: https://www.kaggle.com/datasets/tourist55/alzheimers-dataset-4-class-of-images. License: Open Database License (ODbL) v1.0. [file peerj-cs-10-2459-s002.zip › case1_1_4/test/MildDemented/28 (22).jpg]

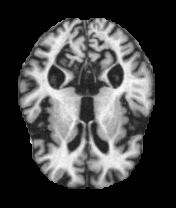

Supplement: Supplemental Information 2 — Image source: https://www.kaggle.com/datasets/tourist55/alzheimers-dataset-4-class-of-images. License: Open Database License (ODbL) v1.0. [file peerj-cs-10-2459-s002.zip › case1_1_4/train/ModerateDemented/moderateDem8.jpg]

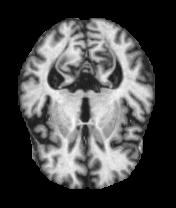

Supplement: Supplemental Information 2 — Image source: https://www.kaggle.com/datasets/tourist55/alzheimers-dataset-4-class-of-images. License: Open Database License (ODbL) v1.0. [file peerj-cs-10-2459-s002.zip › case1_1_4/train/ModerateDemented/moderateDem7.jpg]

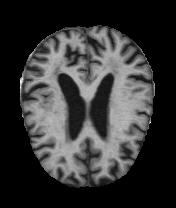

Supplement: Supplemental Information 2 — Image source: https://www.kaggle.com/datasets/tourist55/alzheimers-dataset-4-class-of-images. License: Open Database License (ODbL) v1.0. [file peerj-cs-10-2459-s002.zip › case1_1_4/test/MildDemented/28 (23).jpg]

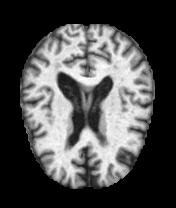

Supplement: Supplemental Information 2 — Image source: https://www.kaggle.com/datasets/tourist55/alzheimers-dataset-4-class-of-images. License: Open Database License (ODbL) v1.0. [file peerj-cs-10-2459-s002.zip › case1_1_4/train/ModerateDemented/moderateDem45.jpg]

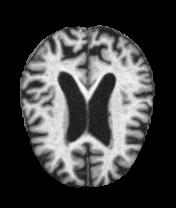

Supplement: Supplemental Information 2 — Image source: https://www.kaggle.com/datasets/tourist55/alzheimers-dataset-4-class-of-images. License: Open Database License (ODbL) v1.0. [file peerj-cs-10-2459-s002.zip › case1_1_4/test/ModerateDemented/28 (2).jpg]

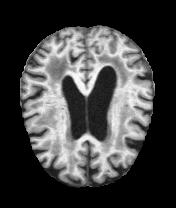

Supplement: Supplemental Information 2 — Image source: https://www.kaggle.com/datasets/tourist55/alzheimers-dataset-4-class-of-images. License: Open Database License (ODbL) v1.0. [file peerj-cs-10-2459-s002.zip › case1_1_4/test/MildDemented/28 (24).jpg]

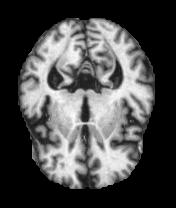

Supplement: Supplemental Information 2 — Image source: https://www.kaggle.com/datasets/tourist55/alzheimers-dataset-4-class-of-images. License: Open Database License (ODbL) v1.0. [file peerj-cs-10-2459-s002.zip › case1_1_4/train/ModerateDemented/moderateDem5.jpg]

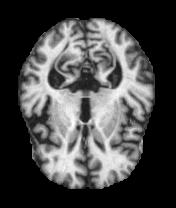

Supplement: Supplemental Information 2 — Image source: https://www.kaggle.com/datasets/tourist55/alzheimers-dataset-4-class-of-images. License: Open Database License (ODbL) v1.0. [file peerj-cs-10-2459-s002.zip › case1_1_4/train/ModerateDemented/moderateDem9.jpg]

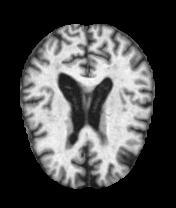

Supplement: Supplemental Information 2 — Image source: https://www.kaggle.com/datasets/tourist55/alzheimers-dataset-4-class-of-images. License: Open Database License (ODbL) v1.0. [file peerj-cs-10-2459-s002.zip › case1_1_4/train/ModerateDemented/moderateDem47.jpg]

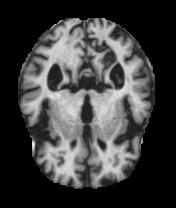

Supplement: Supplemental Information 2 — Image source: https://www.kaggle.com/datasets/tourist55/alzheimers-dataset-4-class-of-images. License: Open Database License (ODbL) v1.0. [file peerj-cs-10-2459-s002.zip › case1_1_4/train/ModerateDemented/moderateDem0.jpg]

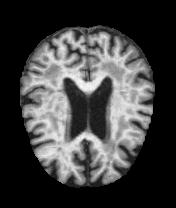

Supplement: Supplemental Information 2 — Image source: https://www.kaggle.com/datasets/tourist55/alzheimers-dataset-4-class-of-images. License: Open Database License (ODbL) v1.0. [file peerj-cs-10-2459-s002.zip › case1_1_4/test/MildDemented/28 (17).jpg]

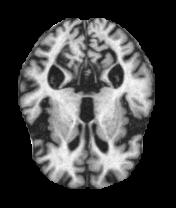

Supplement: Supplemental Information 2 — Image source: https://www.kaggle.com/datasets/tourist55/alzheimers-dataset-4-class-of-images. License: Open Database License (ODbL) v1.0. [file peerj-cs-10-2459-s002.zip › case1_1_4/train/ModerateDemented/moderateDem6.jpg]

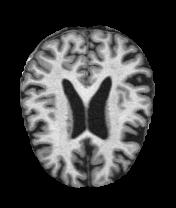

Supplement: Supplemental Information 2 — Image source: https://www.kaggle.com/datasets/tourist55/alzheimers-dataset-4-class-of-images. License: Open Database License (ODbL) v1.0. [file peerj-cs-10-2459-s002.zip › case1_1_4/test/MildDemented/28 (15).jpg]

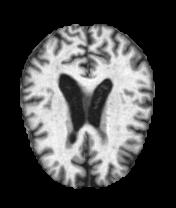

Supplement: Supplemental Information 2 — Image source: https://www.kaggle.com/datasets/tourist55/alzheimers-dataset-4-class-of-images. License: Open Database License (ODbL) v1.0. [file peerj-cs-10-2459-s002.zip › case1_1_4/train/ModerateDemented/moderateDem49.jpg]

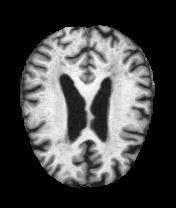

Supplement: Supplemental Information 2 — Image source: https://www.kaggle.com/datasets/tourist55/alzheimers-dataset-4-class-of-images. License: Open Database License (ODbL) v1.0. [file peerj-cs-10-2459-s002.zip › case1_1_4/test/MildDemented/28 (25).jpg]

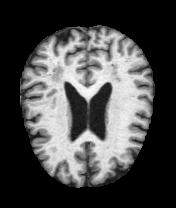

Supplement: Supplemental Information 2 — Image source: https://www.kaggle.com/datasets/tourist55/alzheimers-dataset-4-class-of-images. License: Open Database License (ODbL) v1.0. [file peerj-cs-10-2459-s002.zip › case1_1_4/test/MildDemented/28 (28).jpg]

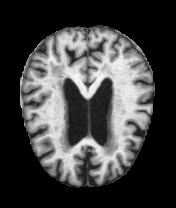

Supplement: Supplemental Information 2 — Image source: https://www.kaggle.com/datasets/tourist55/alzheimers-dataset-4-class-of-images. License: Open Database License (ODbL) v1.0. [file peerj-cs-10-2459-s002.zip › case1_1_4/test/MildDemented/28 (20).jpg]

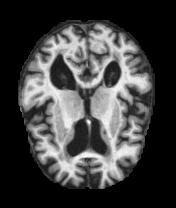

Supplement: Supplemental Information 2 — Image source: https://www.kaggle.com/datasets/tourist55/alzheimers-dataset-4-class-of-images. License: Open Database License (ODbL) v1.0. [file peerj-cs-10-2459-s002.zip › case1_1_4/train/MildDemented/mildDem361.jpg]

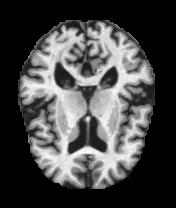

Supplement: Supplemental Information 2 — Image source: https://www.kaggle.com/datasets/tourist55/alzheimers-dataset-4-class-of-images. License: Open Database License (ODbL) v1.0. [file peerj-cs-10-2459-s002.zip › case1_1_4/train/MildDemented/mildDem362.jpg]

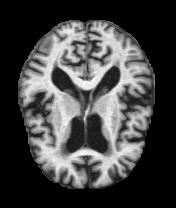

Supplement: Supplemental Information 2 — Image source: https://www.kaggle.com/datasets/tourist55/alzheimers-dataset-4-class-of-images. License: Open Database License (ODbL) v1.0. [file peerj-cs-10-2459-s002.zip › case1_1_4/train/MildDemented/mildDem365.jpg]

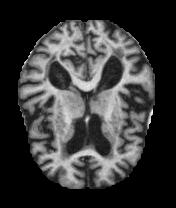

Supplement: Supplemental Information 2 — Image source: https://www.kaggle.com/datasets/tourist55/alzheimers-dataset-4-class-of-images. License: Open Database License (ODbL) v1.0. [file peerj-cs-10-2459-s002.zip › case1_1_4/train/MildDemented/mildDem340.jpg]

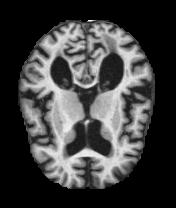

Supplement: Supplemental Information 2 — Image source: https://www.kaggle.com/datasets/tourist55/alzheimers-dataset-4-class-of-images. License: Open Database License (ODbL) v1.0. [file peerj-cs-10-2459-s002.zip › case1_1_4/train/MildDemented/mildDem336.jpg]

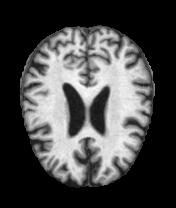

Supplement: Supplemental Information 2 — Image source: https://www.kaggle.com/datasets/tourist55/alzheimers-dataset-4-class-of-images. License: Open Database License (ODbL) v1.0. [file peerj-cs-10-2459-s002.zip › case1_1_4/test/MildDemented/28 (16).jpg]

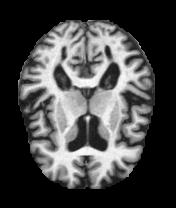

Supplement: Supplemental Information 2 — Image source: https://www.kaggle.com/datasets/tourist55/alzheimers-dataset-4-class-of-images. License: Open Database License (ODbL) v1.0. [file peerj-cs-10-2459-s002.zip › case1_1_4/train/MildDemented/mildDem359.jpg]

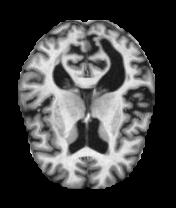

Supplement: Supplemental Information 2 — Image source: https://www.kaggle.com/datasets/tourist55/alzheimers-dataset-4-class-of-images. License: Open Database License (ODbL) v1.0. [file peerj-cs-10-2459-s002.zip › case1_1_4/train/MildDemented/mildDem348.jpg]

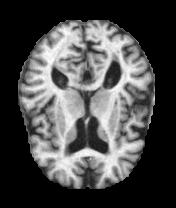

Supplement: Supplemental Information 2 — Image source: https://www.kaggle.com/datasets/tourist55/alzheimers-dataset-4-class-of-images. License: Open Database License (ODbL) v1.0. [file peerj-cs-10-2459-s002.zip › case1_1_4/train/MildDemented/mildDem363.jpg]

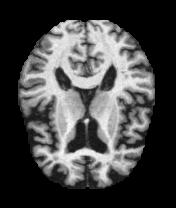

Supplement: Supplemental Information 2 — Image source: https://www.kaggle.com/datasets/tourist55/alzheimers-dataset-4-class-of-images. License: Open Database License (ODbL) v1.0. [file peerj-cs-10-2459-s002.zip › case1_1_4/train/MildDemented/mildDem360.jpg]

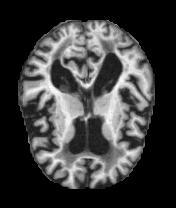

Supplement: Supplemental Information 2 — Image source: https://www.kaggle.com/datasets/tourist55/alzheimers-dataset-4-class-of-images. License: Open Database License (ODbL) v1.0. [file peerj-cs-10-2459-s002.zip › case1_1_4/train/MildDemented/mildDem346.jpg]

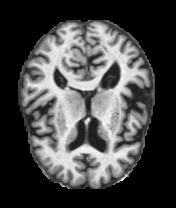

Supplement: Supplemental Information 2 — Image source: https://www.kaggle.com/datasets/tourist55/alzheimers-dataset-4-class-of-images. License: Open Database License (ODbL) v1.0. [file peerj-cs-10-2459-s002.zip › case1_1_4/train/MildDemented/mildDem338.jpg]

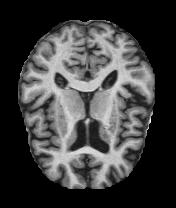

Supplement: Supplemental Information 2 — Image source: https://www.kaggle.com/datasets/tourist55/alzheimers-dataset-4-class-of-images. License: Open Database License (ODbL) v1.0. [file peerj-cs-10-2459-s002.zip › case1_1_4/train/MildDemented/mildDem369.jpg]

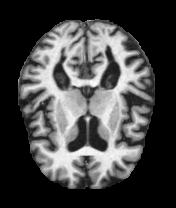

Supplement: Supplemental Information 2 — Image source: https://www.kaggle.com/datasets/tourist55/alzheimers-dataset-4-class-of-images. License: Open Database License (ODbL) v1.0. [file peerj-cs-10-2459-s002.zip › case1_1_4/train/MildDemented/mildDem331.jpg]

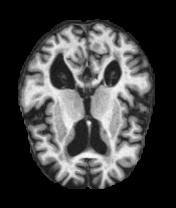

Supplement: Supplemental Information 2 — Image source: https://www.kaggle.com/datasets/tourist55/alzheimers-dataset-4-class-of-images. License: Open Database License (ODbL) v1.0. [file peerj-cs-10-2459-s002.zip › case1_1_4/train/MildDemented/mildDem333.jpg]

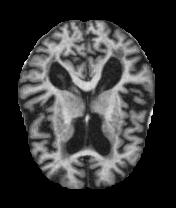

Supplement: Supplemental Information 2 — Image source: https://www.kaggle.com/datasets/tourist55/alzheimers-dataset-4-class-of-images. License: Open Database License (ODbL) v1.0. [file peerj-cs-10-2459-s002.zip › case1_1_4/train/MildDemented/mildDem368.jpg]

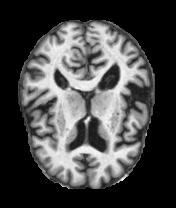

Supplement: Supplemental Information 2 — Image source: https://www.kaggle.com/datasets/tourist55/alzheimers-dataset-4-class-of-images. License: Open Database License (ODbL) v1.0. [file peerj-cs-10-2459-s002.zip › case1_1_4/train/MildDemented/mildDem366.jpg]

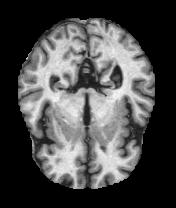

Supplement: Supplemental Information 2 — Image source: https://www.kaggle.com/datasets/tourist55/alzheimers-dataset-4-class-of-images. License: Open Database License (ODbL) v1.0. [file peerj-cs-10-2459-s002.zip › case1_1_4/train/MildDemented/mildDem33.jpg]

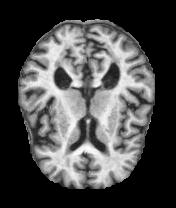

Supplement: Supplemental Information 2 — Image source: https://www.kaggle.com/datasets/tourist55/alzheimers-dataset-4-class-of-images. License: Open Database License (ODbL) v1.0. [file peerj-cs-10-2459-s002.zip › case1_1_4/train/MildDemented/mildDem329.jpg]

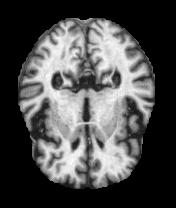

Supplement: Supplemental Information 2 — Image source: https://www.kaggle.com/datasets/tourist55/alzheimers-dataset-4-class-of-images. License: Open Database License (ODbL) v1.0. [file peerj-cs-10-2459-s002.zip › case1_1_4/train/MildDemented/mildDem34.jpg]

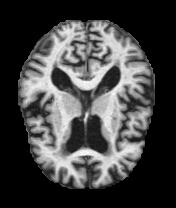

Supplement: Supplemental Information 2 — Image source: https://www.kaggle.com/datasets/tourist55/alzheimers-dataset-4-class-of-images. License: Open Database License (ODbL) v1.0. [file peerj-cs-10-2459-s002.zip › case1_1_4/train/MildDemented/mildDem337.jpg]

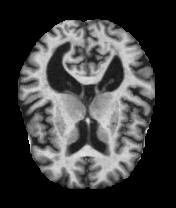

Supplement: Supplemental Information 2 — Image source: https://www.kaggle.com/datasets/tourist55/alzheimers-dataset-4-class-of-images. License: Open Database License (ODbL) v1.0. [file peerj-cs-10-2459-s002.zip › case1_1_4/train/MildDemented/mildDem325.jpg]

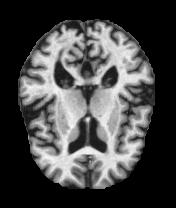

Supplement: Supplemental Information 2 — Image source: https://www.kaggle.com/datasets/tourist55/alzheimers-dataset-4-class-of-images. License: Open Database License (ODbL) v1.0. [file peerj-cs-10-2459-s002.zip › case1_1_4/train/MildDemented/mildDem334.jpg]

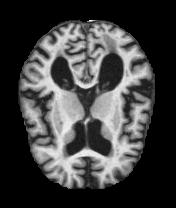

Supplement: Supplemental Information 2 — Image source: https://www.kaggle.com/datasets/tourist55/alzheimers-dataset-4-class-of-images. License: Open Database License (ODbL) v1.0. [file peerj-cs-10-2459-s002.zip › case1_1_4/train/MildDemented/mildDem364.jpg]

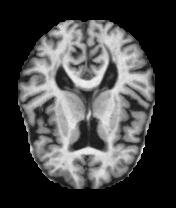

Supplement: Supplemental Information 2 — Image source: https://www.kaggle.com/datasets/tourist55/alzheimers-dataset-4-class-of-images. License: Open Database License (ODbL) v1.0. [file peerj-cs-10-2459-s002.zip › case1_1_4/train/MildDemented/mildDem358.jpg]

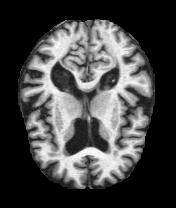

Supplement: Supplemental Information 2 — Image source: https://www.kaggle.com/datasets/tourist55/alzheimers-dataset-4-class-of-images. License: Open Database License (ODbL) v1.0. [file peerj-cs-10-2459-s002.zip › case1_1_4/train/MildDemented/mildDem326.jpg]

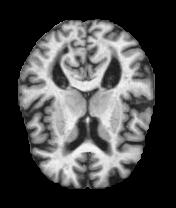

Supplement: Supplemental Information 2 — Image source: https://www.kaggle.com/datasets/tourist55/alzheimers-dataset-4-class-of-images. License: Open Database License (ODbL) v1.0. [file peerj-cs-10-2459-s002.zip › case1_1_4/train/MildDemented/mildDem321.jpg]

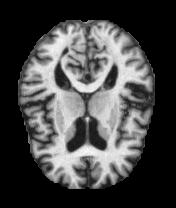

Supplement: Supplemental Information 2 — Image source: https://www.kaggle.com/datasets/tourist55/alzheimers-dataset-4-class-of-images. License: Open Database License (ODbL) v1.0. [file peerj-cs-10-2459-s002.zip › case1_1_4/train/MildDemented/mildDem342.jpg]

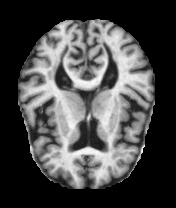

Supplement: Supplemental Information 2 — Image source: https://www.kaggle.com/datasets/tourist55/alzheimers-dataset-4-class-of-images. License: Open Database License (ODbL) v1.0. [file peerj-cs-10-2459-s002.zip › case1_1_4/train/MildDemented/mildDem330.jpg]

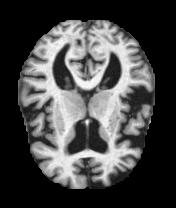

Supplement: Supplemental Information 2 — Image source: https://www.kaggle.com/datasets/tourist55/alzheimers-dataset-4-class-of-images. License: Open Database License (ODbL) v1.0. [file peerj-cs-10-2459-s002.zip › case1_1_4/train/MildDemented/mildDem345.jpg]

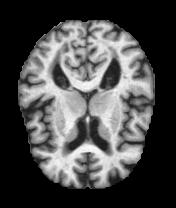

Supplement: Supplemental Information 2 — Image source: https://www.kaggle.com/datasets/tourist55/alzheimers-dataset-4-class-of-images. License: Open Database License (ODbL) v1.0. [file peerj-cs-10-2459-s002.zip › case1_1_4/train/MildDemented/mildDem349.jpg]

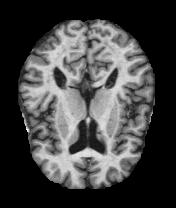

Supplement: Supplemental Information 2 — Image source: https://www.kaggle.com/datasets/tourist55/alzheimers-dataset-4-class-of-images. License: Open Database License (ODbL) v1.0. [file peerj-cs-10-2459-s002.zip › case1_1_4/train/MildDemented/mildDem327.jpg]

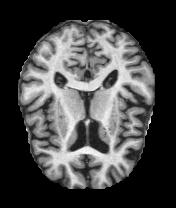

Supplement: Supplemental Information 2 — Image source: https://www.kaggle.com/datasets/tourist55/alzheimers-dataset-4-class-of-images. License: Open Database License (ODbL) v1.0. [file peerj-cs-10-2459-s002.zip › case1_1_4/train/MildDemented/mildDem341.jpg]

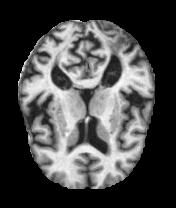

Supplement: Supplemental Information 2 — Image source: https://www.kaggle.com/datasets/tourist55/alzheimers-dataset-4-class-of-images. License: Open Database License (ODbL) v1.0. [file peerj-cs-10-2459-s002.zip › case1_1_4/train/MildDemented/mildDem367.jpg]

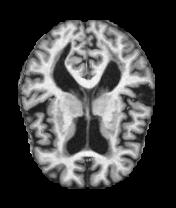

Supplement: Supplemental Information 2 — Image source: https://www.kaggle.com/datasets/tourist55/alzheimers-dataset-4-class-of-images. License: Open Database License (ODbL) v1.0. [file peerj-cs-10-2459-s002.zip › case1_1_4/train/MildDemented/mildDem347.jpg]

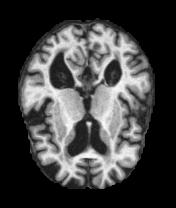

Supplement: Supplemental Information 2 — Image source: https://www.kaggle.com/datasets/tourist55/alzheimers-dataset-4-class-of-images. License: Open Database License (ODbL) v1.0. [file peerj-cs-10-2459-s002.zip › case1_1_4/train/MildDemented/mildDem305.jpg]

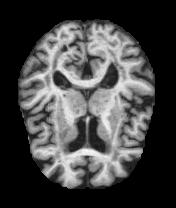

Supplement: Supplemental Information 2 — Image source: https://www.kaggle.com/datasets/tourist55/alzheimers-dataset-4-class-of-images. License: Open Database License (ODbL) v1.0. [file peerj-cs-10-2459-s002.zip › case1_1_4/train/MildDemented/mildDem315.jpg]

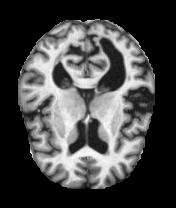

Supplement: Supplemental Information 2 — Image source: https://www.kaggle.com/datasets/tourist55/alzheimers-dataset-4-class-of-images. License: Open Database License (ODbL) v1.0. [file peerj-cs-10-2459-s002.zip › case1_1_4/train/MildDemented/mildDem320.jpg]

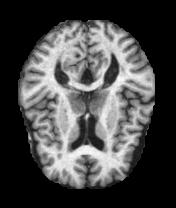

Supplement: Supplemental Information 2 — Image source: https://www.kaggle.com/datasets/tourist55/alzheimers-dataset-4-class-of-images. License: Open Database License (ODbL) v1.0. [file peerj-cs-10-2459-s002.zip › case1_1_4/train/MildDemented/mildDem344.jpg]

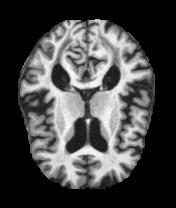

Supplement: Supplemental Information 2 — Image source: https://www.kaggle.com/datasets/tourist55/alzheimers-dataset-4-class-of-images. License: Open Database License (ODbL) v1.0. [file peerj-cs-10-2459-s002.zip › case1_1_4/train/MildDemented/mildDem324.jpg]

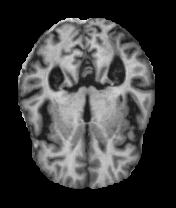

Supplement: Supplemental Information 2 — Image source: https://www.kaggle.com/datasets/tourist55/alzheimers-dataset-4-class-of-images. License: Open Database License (ODbL) v1.0. [file peerj-cs-10-2459-s002.zip › case1_1_4/train/MildDemented/mildDem31.jpg]

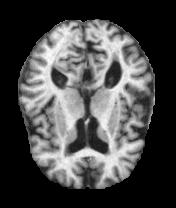

Supplement: Supplemental Information 2 — Image source: https://www.kaggle.com/datasets/tourist55/alzheimers-dataset-4-class-of-images. License: Open Database License (ODbL) v1.0. [file peerj-cs-10-2459-s002.zip › case1_1_4/train/MildDemented/mildDem335.jpg]

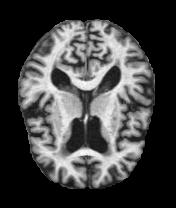

Supplement: Supplemental Information 2 — Image source: https://www.kaggle.com/datasets/tourist55/alzheimers-dataset-4-class-of-images. License: Open Database License (ODbL) v1.0. [file peerj-cs-10-2459-s002.zip › case1_1_4/train/MildDemented/mildDem309.jpg]

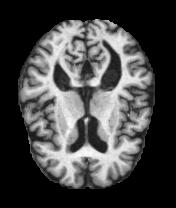

Supplement: Supplemental Information 2 — Image source: https://www.kaggle.com/datasets/tourist55/alzheimers-dataset-4-class-of-images. License: Open Database License (ODbL) v1.0. [file peerj-cs-10-2459-s002.zip › case1_1_4/train/MildDemented/mildDem328.jpg]

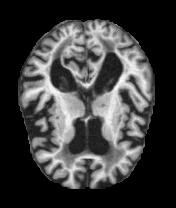

Supplement: Supplemental Information 2 — Image source: https://www.kaggle.com/datasets/tourist55/alzheimers-dataset-4-class-of-images. License: Open Database License (ODbL) v1.0. [file peerj-cs-10-2459-s002.zip › case1_1_4/train/MildDemented/mildDem318.jpg]

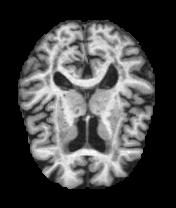

Supplement: Supplemental Information 2 — Image source: https://www.kaggle.com/datasets/tourist55/alzheimers-dataset-4-class-of-images. License: Open Database License (ODbL) v1.0. [file peerj-cs-10-2459-s002.zip › case1_1_4/train/MildDemented/mildDem343.jpg]

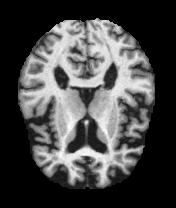

Supplement: Supplemental Information 2 — Image source: https://www.kaggle.com/datasets/tourist55/alzheimers-dataset-4-class-of-images. License: Open Database License (ODbL) v1.0. [file peerj-cs-10-2459-s002.zip › case1_1_4/train/MildDemented/mildDem304.jpg]

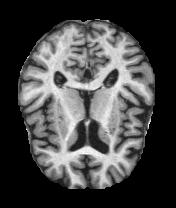

Supplement: Supplemental Information 2 — Image source: https://www.kaggle.com/datasets/tourist55/alzheimers-dataset-4-class-of-images. License: Open Database License (ODbL) v1.0. [file peerj-cs-10-2459-s002.zip › case1_1_4/train/MildDemented/mildDem313.jpg]

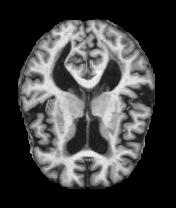

Supplement: Supplemental Information 2 — Image source: https://www.kaggle.com/datasets/tourist55/alzheimers-dataset-4-class-of-images. License: Open Database License (ODbL) v1.0. [file peerj-cs-10-2459-s002.zip › case1_1_4/train/MildDemented/mildDem319.jpg]

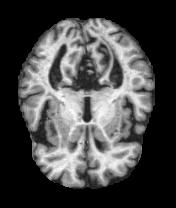

Supplement: Supplemental Information 2 — Image source: https://www.kaggle.com/datasets/tourist55/alzheimers-dataset-4-class-of-images. License: Open Database License (ODbL) v1.0. [file peerj-cs-10-2459-s002.zip › case1_1_4/train/MildDemented/mildDem35.jpg]

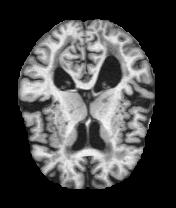

Supplement: Supplemental Information 2 — Image source: https://www.kaggle.com/datasets/tourist55/alzheimers-dataset-4-class-of-images. License: Open Database License (ODbL) v1.0. [file peerj-cs-10-2459-s002.zip › case1_1_4/train/MildDemented/mildDem323.jpg]

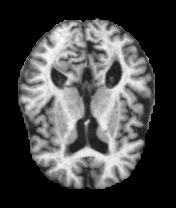

Supplement: Supplemental Information 2 — Image source: https://www.kaggle.com/datasets/tourist55/alzheimers-dataset-4-class-of-images. License: Open Database License (ODbL) v1.0. [file peerj-cs-10-2459-s002.zip › case1_1_4/train/MildDemented/mildDem307.jpg]

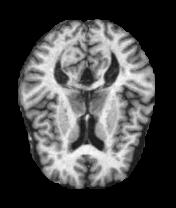

Supplement: Supplemental Information 2 — Image source: https://www.kaggle.com/datasets/tourist55/alzheimers-dataset-4-class-of-images. License: Open Database License (ODbL) v1.0. [file peerj-cs-10-2459-s002.zip › case1_1_4/train/MildDemented/mildDem316.jpg]

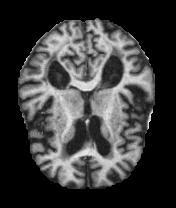

Supplement: Supplemental Information 2 — Image source: https://www.kaggle.com/datasets/tourist55/alzheimers-dataset-4-class-of-images. License: Open Database License (ODbL) v1.0. [file peerj-cs-10-2459-s002.zip › case1_1_4/train/MildDemented/mildDem284.jpg]

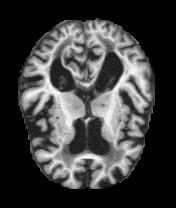

Supplement: Supplemental Information 2 — Image source: https://www.kaggle.com/datasets/tourist55/alzheimers-dataset-4-class-of-images. License: Open Database License (ODbL) v1.0. [file peerj-cs-10-2459-s002.zip › case1_1_4/train/MildDemented/mildDem290.jpg]

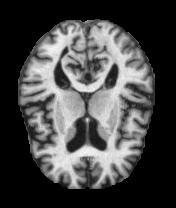

Supplement: Supplemental Information 2 — Image source: https://www.kaggle.com/datasets/tourist55/alzheimers-dataset-4-class-of-images. License: Open Database License (ODbL) v1.0. [file peerj-cs-10-2459-s002.zip › case1_1_4/train/MildDemented/mildDem314.jpg]

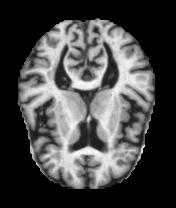

Supplement: Supplemental Information 2 — Image source: https://www.kaggle.com/datasets/tourist55/alzheimers-dataset-4-class-of-images. License: Open Database License (ODbL) v1.0. [file peerj-cs-10-2459-s002.zip › case1_1_4/train/MildDemented/mildDem302.jpg]

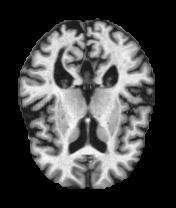

Supplement: Supplemental Information 2 — Image source: https://www.kaggle.com/datasets/tourist55/alzheimers-dataset-4-class-of-images. License: Open Database License (ODbL) v1.0. [file peerj-cs-10-2459-s002.zip › case1_1_4/train/MildDemented/mildDem306.jpg]

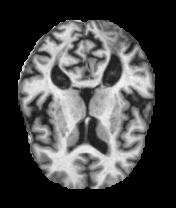

Supplement: Supplemental Information 2 — Image source: https://www.kaggle.com/datasets/tourist55/alzheimers-dataset-4-class-of-images. License: Open Database License (ODbL) v1.0. [file peerj-cs-10-2459-s002.zip › case1_1_4/train/MildDemented/mildDem339.jpg]

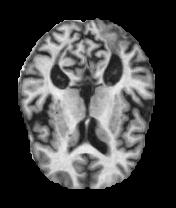

Supplement: Supplemental Information 2 — Image source: https://www.kaggle.com/datasets/tourist55/alzheimers-dataset-4-class-of-images. License: Open Database License (ODbL) v1.0. [file peerj-cs-10-2459-s002.zip › case1_1_4/train/MildDemented/mildDem283.jpg]

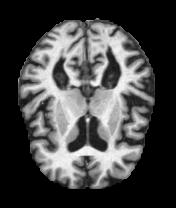

Supplement: Supplemental Information 2 — Image source: https://www.kaggle.com/datasets/tourist55/alzheimers-dataset-4-class-of-images. License: Open Database License (ODbL) v1.0. [file peerj-cs-10-2459-s002.zip › case1_1_4/train/MildDemented/mildDem303.jpg]

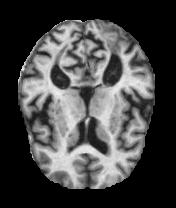

Supplement: Supplemental Information 2 — Image source: https://www.kaggle.com/datasets/tourist55/alzheimers-dataset-4-class-of-images. License: Open Database License (ODbL) v1.0. [file peerj-cs-10-2459-s002.zip › case1_1_4/train/MildDemented/mildDem311.jpg]

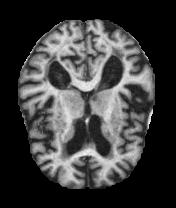

Supplement: Supplemental Information 2 — Image source: https://www.kaggle.com/datasets/tourist55/alzheimers-dataset-4-class-of-images. License: Open Database License (ODbL) v1.0. [file peerj-cs-10-2459-s002.zip › case1_1_4/train/MildDemented/mildDem312.jpg]

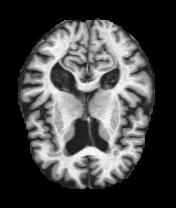

Supplement: Supplemental Information 2 — Image source: https://www.kaggle.com/datasets/tourist55/alzheimers-dataset-4-class-of-images. License: Open Database License (ODbL) v1.0. [file peerj-cs-10-2459-s002.zip › case1_1_4/train/MildDemented/mildDem298.jpg]

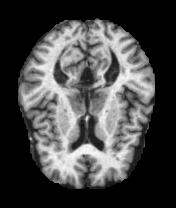

Supplement: Supplemental Information 2 — Image source: https://www.kaggle.com/datasets/tourist55/alzheimers-dataset-4-class-of-images. License: Open Database License (ODbL) v1.0. [file peerj-cs-10-2459-s002.zip › case1_1_4/train/MildDemented/mildDem288.jpg]

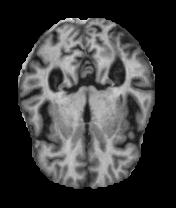

Supplement: Supplemental Information 2 — Image source: https://www.kaggle.com/datasets/tourist55/alzheimers-dataset-4-class-of-images. License: Open Database License (ODbL) v1.0. [file peerj-cs-10-2459-s002.zip › case1_1_4/train/MildDemented/mildDem3.jpg]

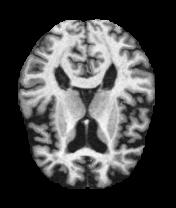

Supplement: Supplemental Information 2 — Image source: https://www.kaggle.com/datasets/tourist55/alzheimers-dataset-4-class-of-images. License: Open Database License (ODbL) v1.0. [file peerj-cs-10-2459-s002.zip › case1_1_4/train/MildDemented/mildDem332.jpg]

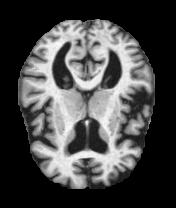

Supplement: Supplemental Information 2 — Image source: https://www.kaggle.com/datasets/tourist55/alzheimers-dataset-4-class-of-images. License: Open Database License (ODbL) v1.0. [file peerj-cs-10-2459-s002.zip › case1_1_4/train/MildDemented/mildDem317.jpg]

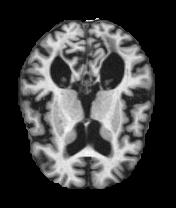

Supplement: Supplemental Information 2 — Image source: https://www.kaggle.com/datasets/tourist55/alzheimers-dataset-4-class-of-images. License: Open Database License (ODbL) v1.0. [file peerj-cs-10-2459-s002.zip › case1_1_4/train/MildDemented/mildDem280.jpg]

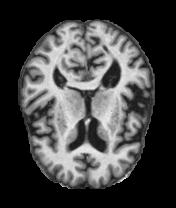

Supplement: Supplemental Information 2 — Image source: https://www.kaggle.com/datasets/tourist55/alzheimers-dataset-4-class-of-images. License: Open Database License (ODbL) v1.0. [file peerj-cs-10-2459-s002.zip › case1_1_4/train/MildDemented/mildDem310.jpg]

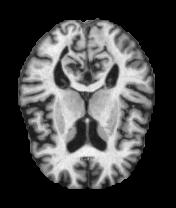

Supplement: Supplemental Information 2 — Image source: https://www.kaggle.com/datasets/tourist55/alzheimers-dataset-4-class-of-images. License: Open Database License (ODbL) v1.0. [file peerj-cs-10-2459-s002.zip › case1_1_4/train/MildDemented/mildDem286.jpg]

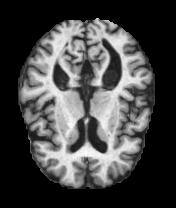

Supplement: Supplemental Information 2 — Image source: https://www.kaggle.com/datasets/tourist55/alzheimers-dataset-4-class-of-images. License: Open Database License (ODbL) v1.0. [file peerj-cs-10-2459-s002.zip › case1_1_4/train/MildDemented/mildDem300.jpg]

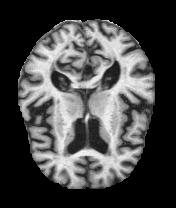

Supplement: Supplemental Information 2 — Image source: https://www.kaggle.com/datasets/tourist55/alzheimers-dataset-4-class-of-images. License: Open Database License (ODbL) v1.0. [file peerj-cs-10-2459-s002.zip › case1_1_4/train/MildDemented/mildDem322.jpg]

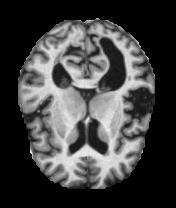

Supplement: Supplemental Information 2 — Image source: https://www.kaggle.com/datasets/tourist55/alzheimers-dataset-4-class-of-images. License: Open Database License (ODbL) v1.0. [file peerj-cs-10-2459-s002.zip › case1_1_4/train/MildDemented/mildDem292.jpg]
